# An Overview of the Recent Advances in Pool Boiling Enhancement Materials, Structrure, and Devices

**DOI:** 10.3390/mi15020281

**Published:** 2024-02-17

**Authors:** José Pereira, Reinaldo Souza, Rui Lima, António Moreira, Ana Moita

**Affiliations:** 1 IN+ Center for Innovation, Technology and Policy Research, Instituto Superior Técnico, University of Lisbon, Avenida Rovisco Pais, 1049-001 Lisbon, Portugal; reinaldo.souza@tecnico.ulisboa.pt (R.S.); aluismoreira@tecnico.ulisboa.pt (A.M.); anamoita@tecnico.ulisboa.pt (A.M.); 2 Mechanical Engineering and Resource Sustainability Center (MEtRICs), Mechanical Engineering Department, University of Minho, Campus de Azurém, 4800-058 Guimarães, Portugal; rl@dem.uminho.pt; 3 Centro de Investigação Desenvolvimento e Inovação da Academia Militar (CINAMIL), Academia Militar, Instituto Universitário Militar, Rua Gomes Freire, 1169-203 Lisboa, Portugal

**Keywords:** pool boiling, electric field, enhanced surfaces, swirl flow, wall deformation

## Abstract

This review attempts to provide a comprehensive assessment of recent methodologies, structures, and devices for pool boiling heat transfer enhancement. Several enhancement approaches relating to the underlying fluid route and the capability to eliminate incipient boiling hysteresis, augment the nucleate boiling heat transfer coefficient, and improve the critical heat flux are assessed. Hence, this study addresses the most relevant issues related to active and passive enhancement techniques and compound enhancement schemes. Passive heat transfer enhancement techniques encompass multiscale surface modification of the heating surface, such as modification with nanoparticles, tunnels, grooves, porous coatings, and enhanced nanostructured surfaces. Also, there are already studies on the employment of a wide range of passive enhancement techniques, like displaced enhancement, swirl flow aids, and bi-thermally conductive surfaces. Moreover, the combined usage of two or more enhancement techniques, commonly known as compound enhancement approaches, is also addressed in this survey. Additionally, the present work highlights the existing scarcity of sufficiently large available databases for a given enhancement methodology regarding the influencing factors derived from the implementation of innovative thermal management systems for temperature-sensitive electronic and power devices, for instance, material, morphology, relative positioning and orientation of the boiling surface, and nucleate boiling heat transfer enhancement pattern and scale. Such scarcity means the available findings are not totally accurate and suitable for the design and implementation of new thermal management systems. The analysis of more than 100 studies in this field shows that all such improvement methodologies aim to enhance the nucleate boiling heat transfer parameters of the critical heat flux and nucleate heat transfer coefficient in pool boiling scenarios. Finally, diverse challenges and prospects for further studies are also pointed out, aimed at developing important in-depth knowledge of the underlying enhancement mechanisms of such techniques.

## 1. Introduction

Pool boiling is an efficient process for heat transfer, mainly because of the associated phase change. With the recent demands of meeting high heat flux dissipation of over 1 kW/cm^2^ in, for instance, electronic chip cooling applications, further enhancement to pool boiling heat transfer is receiving great attention from the research community. In diverse industrial applications, nucleate boiling heat transfer is a surface phenomenon that depends on factors like the heating surface material, morphology, wettability, roughness, porosity, and thermal conductivity. Heat transport is always present in energy harvesting, use, conversion, and recovery processes. Also, the general heat transfer enhancement routes will result in certain improvements in the heat transfer system, which will be more compact and have relevant savings in pumping power and in the overall investment cost. Accordingly, any progress in the nucleate boiling heat transfer capability will entail efficiency improvements and a reduction in the cost of thermal management systems on a large scale. The advantageous feature of phase change heat transfer is its ability to transfer a large amount of energy in the form of heat. Also, the boiling process is an efficient heat transfer process due to its high latent heat and is usually applied in processes where high heat fluxes must be dissipated, including nuclear reactors [1]; desalination systems [2]; cooling of electronics [3]; and thermal management systems, including devices like heat pipes [4], heat exchangers [5], and thermosyphons [6]. Pool boiling heat transfer is associated with transient conduction, microlayer evaporation, and micro-convection. Such mechanisms are closely linked to the liquid–vapor interface around a nucleating, growing, and departing vapor bubble on the heating surface. The wettability of the liquid–vapor interface determines the shape of the interface, the motion of the contact line, and related processes. The advances related to surface energy modification of the heating surface have paved the way for enhancing the nucleate pool boiling heat transfer by altering the contact angle through the incorporation of nanostructured features and layers on the surface. These nanostructures alter the wettability and improve the critical heat flux (CHF) of the heat transfer surface. The enhancement of the nucleate boiling heat transfer behavior encompasses several goals, including initiating nucleate boiling (incipient boiling) at lower heat fluxes and wall superheats, preventing or mitigating the temperature excursion and sharp temperature drop inherent to the boiling incipience, reducing the wall superheats by increasing the number of active nucleation sites and bubble departure frequency, and delaying the CHF to higher heat fluxes. The employment of enhanced structured boiling surfaces can be a promising route to increase the available heat transfer surface area, disrupt the coalescent bubbles, and improve the CHF of the system. The pool boiling enhancement process is usually applied to achieve the following objectives: (i) initiation of the nucleate boiling at a lower boiling surface temperature; (ii) mitigation or elimination of the incipience temperature excursion; (iii) reduction in the temperature of the heat transfer surface, i.e., increase in the heat transfer coefficient (HTC) associated with nucleate boiling; and (iv) increase in the CHF value to support higher surface heat fluxes. The heat is transferred from the heat transfer surface to the operating fluid during the nucleate boiling process, and the heat transfer is carried out by convection because of the motion of the fluid. Also, the surface tension and density gradient, which produce the buoyancy force, are important parameters, together with the latent heat. The buoyancy flow and latent heat of the boiling heat transfer process result in higher heat transfer rate and coefficient when compared to single-phase convection [7]. The main beneficial features offered by a boiling enhanced curve when compared to a typical boiling curve corresponding to a heating surface without modification are the increase in CHF and the lower onset of nucleate boiling, meaning that in the enhanced curve boiling process, there is a need for lesser thermal loads to initiate nucleate boiling when compared to the loads needed in the boiling curve boiling process. In addition, it can be emphasized that thermal management heat transfer has attracted great attention from the research community dealing with the development and implementation routes for pool boiling heat transfer amelioration. One of the most commonly followed routes, among the others described in this work, like displaced heat transfer enhancement and the use of improved thermal fluids, is boiling surface modification. This approach directly deals with the inclusion in the heat transfer surface of structures at the nano/microscale [8], including porous coatings, nanoparticles [9], nanofibers, nanotubes covering, and mixed wettability characteristics [10,11]. Usually, physical [12] and chemical [13] methodologies are employed to process the intended heating surface modification, along with the exploration of a wide range of different materials, such as metals [14], metal oxides [15], ceramics [16], carbon [17], graphene, and graphene oxide [18]. The methodologies include physical vapor deposition (PVD), all types of chemical vapor deposition (CVD), electrochemical etching, spraying, magnetron sputtering, spin coating, plasma coating, and sintering, among many others. Generally, the investigation pathways require that the developed enhanced nucleate boiling heat transfer surfaces be subjected to comparison with plain, bare heating surfaces in terms of heat transfer capability, which is translated in their intrinsic parameters, like HTC and CHF. Published studies on the matter reveal that the adoption of modified enhanced boiling surfaces directly influences the nucleate HTC and CHF values at pool boiling scenarios, but normally the interpretation of the underlying mechanisms that provoke such an impact varies from researcher to researcher rather than being totally reliable and consistent [19]. For instance, different researchers have studied the vapor bubble dynamics [20,21] and deduced that the combined effect of the adhesion, buoyancy, and surface tension forces present in modified heating surfaces induced the merging of the vapor bubbles. Other authors have confirmed that the total number of vapor bubbles in a structured enhanced surface is much greater than that provided by a bare, plain boiling surface at an imposed heat flux. Moreover, other authors have stated that the heat-transfer-enhancing effect of modified porous heating surfaces come from their improved wettability character, closely linked to the capillary effect [22,23]. Moreover, it can be noted that the most commonly encountered mechanisms responsible for the enhancement of boiling heat transfer can be various, with improved surface wettability, mixed wettability, hydrodynamic instability, modified average surface roughness, and wickability action, among others, coming into play at diverse stages of the nucleate boiling process. The main goal is always to develop and implement a boiling system with a heating surface with improved heat transfer behavior and better heat dissipation capability. The following sections will summarize the main passive pool boiling heat transfer enhancement techniques available, together with a brief explanation of their boiling driving mechanisms at different stages. Additionally, the combined employment of two or more heat transfer enhancement techniques designated by compound enhancement techniques is also discussed in the present study. Finally, some of the main limitations, challenges, and prospects for further investigation in the field of boiling thermal management systems are pointed out in Section 3 of this work.

## 2. Pool Boiling Enhancement Techniques

**Figure 1:**
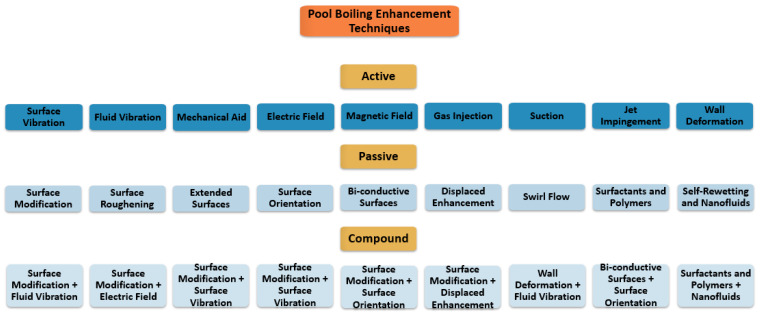
Main pool boiling enhancement techniques.

The available techniques for nucleate boiling heat transfer improvement can be classified into active, passive, and hybrid or compound enhancement techniques, as shown in the diagram in Figure 1. The active techniques involve technical strategies like heating surface vibration, fluid vibration and suction, gas injection, and the application of external electric or magnetic fields for heat transfer enhancement purposes. However, the active techniques are relatively expensive and hard to apply in compact cooling enclosures, such as those used in the cooling of electronics. The passive techniques encompass various procedures of surface modification; the use of enriched working fluids and additives; and refined working conditions, like heating transfer surface orientation adjustment, pool boiling confinement, and liquid pool height. The hybrid or compound enhancement techniques involve one active technique coupled with one passive technique or, alternatively, the combined usage of two or more active enhancement techniques.

### 2.1. Active Techniques

#### 2.1.1. Surface Vibration

**Figure 2:**
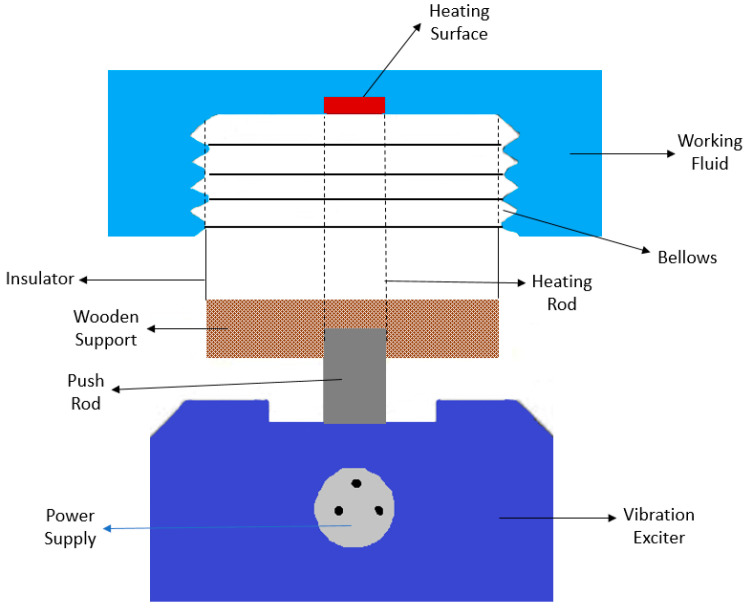
Schematic diagram of a surface vibration apparatus. Adapted from [33].

Several published studies have already demonstrated that applying high-frequency and high-amplitude oscillations to the boiling surface may lead to nucleate boiling heat transfer enhancement [24,25,26,27]. Also, it can be assumed that combinations of frequency and vibration amplitude of the heating surface may lead up to a 2-fold increase in the HTC value. Sufficiently intense oscillations can improve the heat transfer capability of the operating fluids by applying a surface vibration with a frequency lower than 1000 Hz. The vibration of the boiling surface through the action of an electrodynamic vibrator or motor-driven eccentric will break the boundary layer and move the nanoparticles dispersed in the fluid over to the nearby boiling surface region. Such an effect can induce forced convection in a free convection region. Additionally, the researchers Prisnyakov et al. [28] have already demonstrated that the value of heat fluxes removed from a vibrating surface strongly depends on the presence or absence of boiling on such a surface. The authors observed that in the convection region, an increase in the thermal load decreased the nucleate boiling heat transfer performance and vice versa. The authors obtained a 2-fold increase in the heat transfer coefficient with a vibrating heating surface, and that increase was directly proportional to the frequency and amplitude of the vibrations. Also, the researchers Zitko and Afgan [29] investigated the heat transfer performance of water pool boiling with a vibrating heating surface. In their experiments, the imposed heat flux varied from 0 to 85 × 10^−4^ W/m^2^. The authors also varied the vibration amplitude between 0.1 and 2 mm and their frequency from 0 to 70 Hz. Based on the obtained results, the authors concluded that the HTC increased with increasing heat flux, vibration frequency, and vibration amplitude. Furthermore, the researchers Atashi et al. [30] investigated the impact of low-frequency vibrations on the boiling heat transfer capability. The authors stated that the vibrations generated turbulence and extra nucleation sites, leading to the nucleation of bubbles with smaller sizes when compared to those nucleated on a non-vibrating heating surface. The main result was achieved at a frequency of 25 Hz, where the heat transfer improved by up to 116.6% under low-frequency vibrations. In addition, the authors Sathyabhama et al. [31] investigated the effects of mechanical vibration on the pool boiling process using a smooth copper surface. The authors observed an HTC increase at low vibration frequency and amplitude conditions. On the contrary, the researchers also found that the HTC deteriorated at higher vibration amplitudes and frequencies. The HTC was found to be enhanced by up to 26% with increasing mechanical vibration intensity. Moreover, the authors Abadi et al. [32] studied water pool boiling at atmospheric pressure with the assistance of the vertical vibration of an array of heating tubes. The obtained results revealed that the vibration had considerable effect because it enhanced the HTC by up to around 90%. The researchers also observed that the effect of the vibration of the heat transfer tubes was more prominent under low imposed heat fluxes. Also, in the experimental work carried out by the authors Alangar et al. [33], the impact of the vertical vibration of a copper heating surface on its water nucleate pool boiling at atmospheric pressure was inferred. The researchers varied the vibration frequency between 0 and 25 Hz and the amplitude of vibration between 0 and 5 mm. The experimental results indicated that the surface vibration enhanced the nucleate boiling heat transfer capability of the system. It was concluded that the effect of the vertical vibration of the boiling surface was significant at low heat fluxes. The authors also observed that the heat flux that can be removed from the heating surface at a given temperature increased with the increasing intensity of the vibration. The gain in heat transfer behavior was attributed by the authors to changes in the vapor bubble parameters. The experimental data were consistent with those published for high heat fluxes. The schematic diagram of the laboratorial set-up employed in this experiment is presented in Figure 2. Moreover, the authors Alimoradi et al. [34] numerically investigated the pool boiling process using a silicon oxide nanofluid as operating fluid and a vibrating heat transfer surface. To consider the heat dissipation caused by the vapor bubble nucleation and departure, the authors employed the Rensselaer Polytechnic Institute (RPI) boiling method and arrived at the following main conclusions: (i) the heating surface vibration augmented the pool boiling heat transfer capability, and this effect was most remarkable at low imposed heat fluxes, regardless of the amplitude and frequency of vibration; (ii) the increase in the amplitude of vibration stimulated an increase in heat transfer rate of nearly 30.1% at an imposed heat flux of 10.9 kW/m^2^ and of around 3.9% at an imposed heat flux of 265.5 kW/m^2^, with Y_wall_ = 3 mm and f_vib_ = 10 Hz, compared to a non-vibrating surface; (iii) the amplitude of vibration slightly improved the heat transfer capability compared to the frequency of vibration; and (iv) the HTC increased with increasing nanofluid concentration.

#### 2.1.2. Fluid Vibration

**Figure 3:**
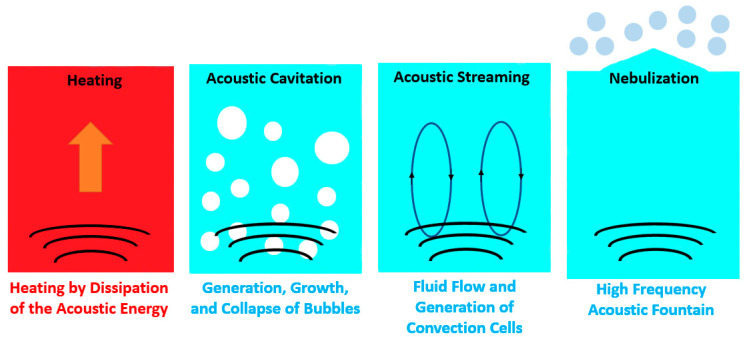
Schematic diagram representing the ultrasound-assisted pool-boiling-related phenomena. Adapted from [40].

Among the active fluid vibration heat transfer enhancement techniques, the application of an ultrasonic field in the boiling fluid has emerged as the most commonly employed fluid vibration technique for nucleate boiling heat transfer improvement, as has already been demonstrated by several researchers. The ultrasonic waves induce a force field into the boiling process, which affects the entire mechanism from nucleation to the subsequent stages of growth and collapse of the bubbles in the fluid [35]. An ultrasonic probe can create an acoustic wave field in the fluid that induces volume oscillations and surface waves on the bubbles, which facilitate their departure. Also, the bubbles tend to coalesce at higher heat fluxes, which leads to vapor film formation over the heating surface. The ultrasonic waves delay the vapor film production, leading to enhanced heat transfer capability. During the boiling process, the buoyancy force plays a prominent role in nucleate boiling by promoting the departure of the bubbles from the surface. The bubble removal is impounded in areas with an absence of gravity, and this may lead to the blanketing of the heating surface with a vapor film and deterioration of the heat transfer. Also, it should be noted that inefficient film boiling heat transfer induces high surface temperatures, leading to an eventual burnout of the boiling surface. The ultrasonic waves may provide a suitable replacement for the gravity effect to maintain stable nucleate boiling [36]. An ultrasonic field creates spatial pressure variations, and when the local pressure falls below the vapor pressure, the stretching of the fluid leads to the formation of vapor-filled cavities, usually designated as cavitation bubbles. Acoustic cavitation can be defined as the formation of bubbles due to the volumetric oscillations of pressure and their subsequent growth and collapse within the fluid. Such mechanisms stimulate the agitation of the working thermal fluid, assist in the disruption of the stagnant film at the heating surface, and, hence, lead to an improved nucleate boiling heat transfer rate. Figure 3 illustrates the general principles of the ultrasound-assisted pool boiling process. Furthermore, the researchers Baffigi and Bartoli [37] explored 25 and 35 °C subcooling in nucleate pool boiling under the influence of an ultrasonic field with 38 kHz frequency. It was found that HTC was enhanced by 45% for subcooling of 35 °C at a heat flux of 1.4 × 10^5^ W/m^2^, whereas a nearly 26% enhancement was reported for 25 °C subcooling at a heat flux of 1.0 × 10^5^ W/m^2^ for a maximum ultrasonic power of 500 W. The obtained experimental results also exhibited a nucleate boiling heat transfer enhancement with increasing ultrasonic generator power. An increase of 114% in the HTC was reported at a power of 500 W and 25 °C subcooling, whereas the corresponding HTC enhancements for 300 and 400 W were 66% and 90%, respectively. Also, the authors interpreted the results based on the ease of bubble detachment provided by the ultrasonic field, along with the increase in subcooling degree. Furthermore, the researchers Tang et al. [38] evaluated the effect of an ultrasonic field of 20 kHz on vapor bubbles in subcooled boiling and reported an improvement in the Nusselt number and easier collapse of the bubbles under ultrasonic field action. The authors concluded that the ultrasonic field makes the thermal boundary layer unstable near the bubbles, and the formation of capillary waves stimulates the condensation of the bubbles, leading to their collapse. Additionally, the researchers Bartoli and Baffigi [39] evaluated the impact of the positioning of the heating surface on nucleate boiling heat transfer enhancement under the action of an ultrasonic field. The authors found that the ideal surface location was at a distance of 50 mm from the wall and 15 mm above the bottom of the pool boiling tank with an imposed heat flux of 1.2 × 10^5^ W/m^2^ and an ultrasound frequency adjusted to 40 kHz. Also, the authors stated that the diverse heating surface morphologies and possible dimensions had a considerable influence on the ultrasound-assisted nucleate boiling heat transfer process.

Additionally, the researchers Hetsroni et al. [41] investigated the impact of the size of the heating surface on the effect of an ultrasonic field. The investigation team studied the effect of wires with sizes ranging from 20 to 250 mm and concluded that the effect of the ultrasonic field on the pool boiling process was dependent on the dimension of the heating surface. In the case of using the smallest wire with 20 mm, no improvement was observed because of the resulting less-strong vapor jets. The reduction in the wall temperature and the improvement in the boiling heat transfer capability were more pronounced in cases where larger wire sizes were employed. The maximum reduction in the wall temperature was reported using the 0.2 mm sized heating wire, whereas the HTC was enhanced by around 45%. The obtained results were interpreted by the authors based on the increased turbulence, wall shear force, and secondary acoustic streaming. The investigation of pool boiling under an ultrasonic field and different gravity conditions was introduced by the researchers Sitter et al. [42], who studied the effect of the acoustic field on the nucleate pool boiling process under terrestrial and microgravity conditions using a frequency of around 10.2 kHz and an acoustic pressure of 260 kPa. Under microgravity, the acoustic wave field force facilitated the detachment of the bubbles from the heating surface because the buoyancy force was absent. These bubbles, along with the vapor bubbles, created microagitation in the fluid and contributed to heat transfer enhancement. Additionally, Kim et al. [43] investigated the impact of the cavitation process on the natural convection and nucleate boiling regimes under the effect of an ultrasonic field. In the natural convection regime, both the mobility and density of the cavitation bubbles contributed appreciably to heat transfer augmentation. However, in the subcooled and saturated boiling regime, regardless of the reduction in the departure diameter and increase in the frequency and mobility of the vapor bubbles, the enhancement ratio of the heat transfer capability was strongly reduced. Because the ultrasonic waves are relatively low-energy waves and get easily damped, their remarkable effectiveness in assisting the boiling heat transfer process is derived only in the case of employing resonance or high generator ultrasonic power. The impact of the subcooling degree, operating pressure, boiling surface morphology, and the relative placement within the ultrasonic field should be carefully considered to infer the performance of boiling heat transfer under the action of an ultrasonic field. This need for accurate evaluation is one of the major reasons behind the only minor progress made in the research field of ultrasonic-assisted pool boiling. Nonetheless, the following conclusions can be noted: (i) The ultrasonic wave propagation in the pool boiling process affects the nucleation, growth, detachment, and motion of the vapor bubbles through the fluid. (ii) The surface modification involving structured surfaces (e.g., micro-channeled/finned surfaces) has been found to be favorable to nucleate pool boiling under the influence of an ultrasonic field. A decrease in the superheat degree and an increase in the HTC has been found for diverse enhanced boiling surfaces. (iii) The relative positioning of the heating surface in the ultrasonic field has been proved to influence the nucleate boiling heat transfer performance. (iv) Under microgravity, the bubble detachment from the heating surface becomes more difficult because of the insufficient buoyancy force, and the ultrasonic waves stimulate the displacement of the vapor bubbles. (v) Nucleate boiling heat transfer improvement under the action of an ultrasonic field is dependent on the frequency of the field, and this experimental parameter must be chosen in such a way that enables the bubble equilibrium radius to be kept near its departure radius to ensure high heat and mass transfer rates and facilitate the bubble departure stage. Nevertheless, a higher ultrasonic power has been found to foster ultrasonic pool boiling heat transfer enhancement caused by an increase in acoustic pressure. (vi) When dealing with saturated boiling cases, the ultrasonic field effect is not so effective because of the increased attenuation from the vapor bubbles at higher imposed heat fluxes. However, subcooling between 15 and 35 °C improves heat transfer behavior in the ultrasonic-assisted pool boiling process. The diverse types of surfaces, such as hydrophilic and hydrophobic types, and coatings are possible technical solutions that need to be further studied under the action of an ultrasonic field. Nonetheless, there is still a lack of rigorous numerical simulations and parametric studies, such as further investigations on the possible application of variable pressure along with different subcooling in the ultrasonic field nucleate pool boiling process. Meanwhile, it should be emphasized that it is not only ultrasonic waves that are applied in active pool boiling heat transfer enhancement techniques of fluid vibration. For instance, the numerical work performed by the researchers Mondal and Bhattacharya [44] evaluated the impact of induced vibrations in the operating fluid on the pool boiling heat transfer performance amelioration. In this direction, the authors adopted the single-component multiphase relaxation-time-based Lattice Boltzmann method (LBM) and modulated the ebullition cycles of the vapor bubbles from single and multiple nucleation sites with different nucleation densities in a pool with fluid motion provided by moving solid boundaries that periodically moved at a given frequency and amplitude values. The results provided useful insights into the nucleation, growth, and detachment stages of bubbles in stationary and fluid-motion conditions. It was found that the vibration in the fluid enhanced the growth rate and bubble departure frequency of the bubbles because of the additional forces acting on them, which facilitated their growth and detachment. Also, the surface heat flux was found to be significantly higher for the moving solid boundaries at a given boiling surface superheat value. The referred study on the motion frequency and amplitude of the solid boundaries indicated that there were ideal frequency and amplitude values that made the bubble departure frequency reach a maximum, and beyond such values, the bubble departure frequency decreased.

#### 2.1.3. Mechanical Aid

**Figure 4:**
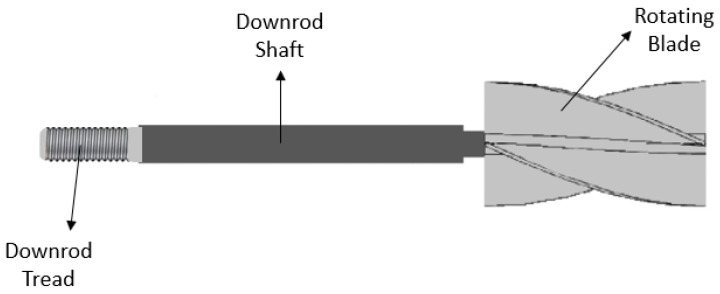
Scheme of a four-blade rotating blade. Adapted from [45].

Some researchers have been aiming to enhance the nucleate boiling heat transfer capability through the creation of strong fluid motion disturbance over the heating surface with the aid of custom-developed mechanical parts and/or devices. In this direction, the experimental work performed by the authors Suriyawong et al. [45] analyzed the nucleate pool boiling heat transfer of water with the assistance of copper rotating blades over a copper heating surface. The blades had a diameter of 30 mm, a core of 5 mm, a length of 50 mm, and a blade angle of 90°. The authors used two, three, and four blades and distances between the surface and the tip of the blades of 5, 15, and 25 mm. Figure 4 schematically represents the four-blade rotating blade that was used in the experiments.

**Figure 5:**
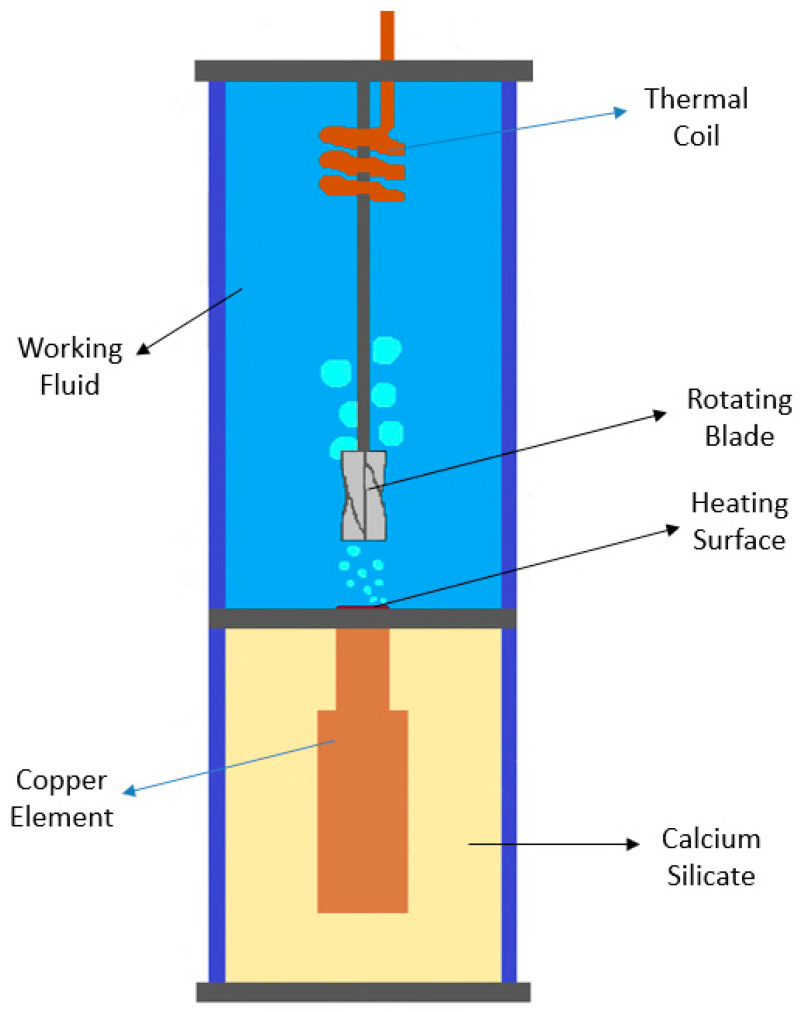
Schematic diagram of the set-up with a four-blade rotating blade. Adapted from [45].

The obtained results indicated that a decrease in the distance between the surface and the tip of the blades enhanced the HTC. It was also found that the technical solution employing four blades placed at 5, 15, and 25 mm from the heating surface yielded the highest HTC enhancements of nearly 29.7%, 18.6%, and 12.4%, respectively. The fact that the 5 mm distance promoted the highest HTC enhancement was explained by the authors as being the result of the increased likelihood of vapor bubbles striking the rotating blades in the shortest distance, creating more fluid motion disturbance over the boiling surface. Additionally, the results also revealed that increasing the number of employed blades promoted HTC enhancement. For instance, at 5 mm from the surface, the HTC improvement with two, three, and four blades was around 13%, 19.8%, and 29.7%, respectively. The authors interpreted these results based on the fact that the added blades increased the area that received the strike force from the bubbles, and as a result, the rotating blades created more disturbance in the working fluid motion over the boiling surface. Figure 5 presents the schematic diagram of the set-up of the experiments.

Another case of a practical methodology that generates fluid disturbance is the one proposed by the researchers Ashouri et al. [46], who placed an inner corrugated hollow conical frustum (ICHCF) above the heat transfer surface to improve the thermal performance of the pool boiling process. The authors evaluated the behavior of various ICHCFs in both stationary and rotating cases, and the impact of various factors, including the height and thread depth of the ICHCF, distance between the ICHCF and the boiling surface, rotational velocity of the ICHCF, and temperature increase, were investigated. In view of the obtained results, the authors arrived at the following main conclusions: (i) the increase in the height of the ICHCF enhanced the HTC in both rotating and stationary modes; (ii) the study found an ideal imposed heat flux in the rotating mode at which the maximum HTC value was achieved; (iii) for both rotating and stationary modes, the excess temperature decreased with increasing ICHCF height; (iv) the rotating ICHCF enhanced the HTC regardless of its height and imposed heat flux; (v) the rotational speed increase in the ICHCF augmented the HTC, but after a certain speed value, the enhancement decreased with increasing speed; (vi) the smaller pitches and higher thread depths improved the HTC; and (vii) HTC enhancements of up to 19.8% and 1302% were obtained in the stationary and rotating modes, respectively, compared to those obtained using a plain surface.

#### 2.1.4. Electric Field

**Figure 6:**
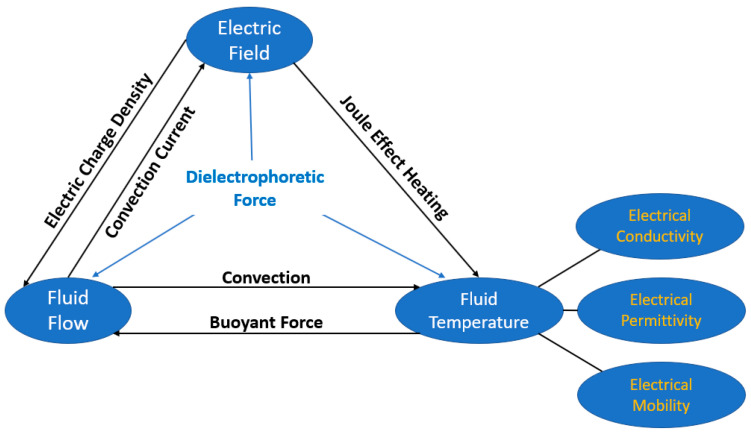
Schematic diagram of the electrohydrodynamic (EHD) interactions. Adapted from [51].

The application of an external electric field has already been demonstrated to be a very suitable methodology to enhance the nucleate boiling heat transfer performance, especially by breaking the bubbles and preventing dry-out. In this sense, the researchers Zaghdoudi and Lallemand [47] studied pool boiling heat transfer enhancement by a DC electric field for n-pentane, R-113, and R-123. The authors found that high DC electric field promoted HTC and CHF enhancements and prevented hysteresis. The level of enhancement of the HTC and CHF through the action of the electric field varied with different working fluids employed in the experiments. Accordingly, the authors Hristov et al. [48] observed a similar phenomenon through the nucleate pool boiling process of the R-123 thermal fluid under an electric field. The authors also found that their results were not consistent with the published ones from similar experiments but under different surface conditions and different wire mesh electrode configurations. Additionally, the authors Di Marco and Grassi [49] studied the enhancement in nucleate pool boiling induced by the electric field under both terrestrial and microgravity conditions. It is well known that there is an additional force acting on the vapor bubbles under the influence of an electrostatic field, given that the electric permittivity of the vapor is different from that of the fluids. Moreover, other researchers have investigated the effect of the electric field acting together with different types of structures. This was the case with the authors Darabi and Ekula [50], who developed a chip-integrated microcooling device by combining electrohydrodynamic (EHD) pumping to form a thin film and used its evaporation to dissipate heat. The fundamental EHD interactions are summarized in the schematic diagram in Figure 6.

#### 2.1.5. Magnetic Field

**Figure 7:**
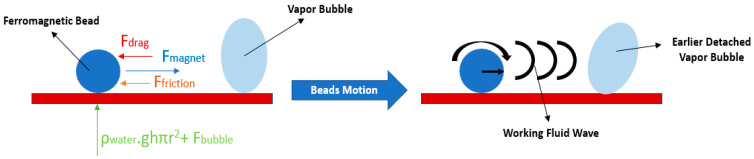
Schematic diagram of the actuating forces and principle of actuation of the beads. Adapted from [53].

Magnetic fields have already been applied to enhance boiling heat transfer. These fields improve the heat transfer from the boiling surface to the fluid by creating external forces. Boiling heat transfer enhancement under the influence of a magnetic field is derived mainly from the effect of the magnetic field on the vapor bubbles. When a non-uniform magnetic field is applied, the magnetic thermal fluids will be under the action of the magnetic body force, which is directly proportional to the magnetization magnitude. The magnetization of a magnetic fluid decreases with its increasing temperature, and hence, it is larger in the lower temperature layer where the magnetic body force is stronger. The magnetic fluids can be attracted by the magnetic body force in the high magnetic field strength region, while the bubbles in the magnetic fluid are transferred from the strong magnetic force regions to the weak magnetic force regions. The force pointing toward the low magnetic field strength is called magnetic levitation force and acts on the vapor bubbles. At the departure stage of the bubbles from the heating surface and without the magnetic field activation, the buoyancy force can be balanced by the surface tension. In cases where magnetic field is applied, the magnetic levitation force acting on the bubble gains importance. In cases where the magnet is placed at the bottom of the pool boiling vessel, there exists an equilibrium of forces on the bubble, that is, the upward magnetic levitation force results in a smaller bubble departure diameter. Furthermore, under an applied magnetic field, a force will act at the center of the vapor bubbles, making them elongated and aligned with the direction of the magnetic field. The heat is transmitted from the heating surface to the bubbles by the evaporation of the superheated layer between the bottom of the bubbles and the surface. Also, the magnetization in the temperature difference layer is weaker than in the bulk liquid, resulting in a weaker magnetic force so that the elongated bubble deforms further as its bottom spreads out on the heating surface. Thus, the bubble becomes slender in the middle and broader at the bottom, and this new shape increases the area of the temperature difference layer beneath the bottom of the bubble and causes faster growth of the bubbles by the evaporation of the microlayer and a higher lift-off speed. Though the bubbles exhibit smaller departure diameters, faster growth, and higher velocities in the presence of a magnetic field, the lower number of bubbles generated stimulates only a minor improvement in the nucleate boiling heat transfer performance at low heat fluxes, where the natural convection boiling surface area is larger. At high heat fluxes, the nucleate boiling surface area also increases with the presence of more bubbles on the surface, and the influence of the magnetic field on the bubbles in the boiling heat transfer becomes stronger and, consequently, the boiling heat transfer enhancement increases remarkably. An example of a published work on the matter is that of Ozdemir et al. [52], who enhanced nucleate boiling heat transfer by nearly 42% using magnetic nanoparticles dispersed in water as operating fluid and with the assistance of a magnetic field. The authors reported that in cases where nanoparticles with higher mass ratios were suspended and with no application of the magnetic field, more vapor bubbles gathered on the heating surface, diminishing the heat transfer capability. The researchers interpreted this fact based on the increase in fluid drag caused by the rising bubbles due to the mass fraction of the magnetic nanoparticles. Nevertheless, under the effect of the magnetic field and because of the improved mixing of nanoparticles, the influence of the mass fraction on the nucleate boiling heat transfer performance became negligible. The authors Rahmati et al. [53] investigated water nucleate boiling using a copper heating surface covered by a mixture of magnetic and non-magnetic beads under the action of an alternating magnetic field. The forces acting on the beads and the principles of actuation of the beads for heat transfer performance enhancement are presented schematically in Figure 7. The number of beads was selected in such a way that they would cover 1/3, 1/2, and 2/3 of the heating surface. The magnetic field was adjusted through the coil’s input voltage. It was found that the beads increased the number of nucleation sites as they extended the available heat transfer surface and improved the mixing because of the random movement of the beads on the surface. Also, when the magnetic field was applied, the movement of the beads became more organized, and the bubble detachment rate suddenly increased because of the stronger force generated from the beads. In view of the obtained experimental results, the authors arrived at the following conclusions: (i) There was an optimum number of beads that maximized the HTC. An excessive number of beads hindered their movement and eliminated the influence of the applied magnetic field. An insufficient number of beads reduced the available heat transfer area and collisions between the beads. (ii) In cases where the magnetic field was not applied, the HTC value did not vary appreciably with the different number of beads. Nonetheless, in cases where the magnetic field was applied, the HTC increased, which was caused by the vigorous mixing promoted by the motion of the beads and the consequent turbulence. In these last cases, the nucleate HTC was enhanced with increasing field strength, with the highest enhancement reported for the application of a magnetic field of 90 V, where the HTC value was 22% higher than that obtained without magnetic field action. (iii) The nucleate boiling heat transfer performance was ameliorated with increasing magnetic field cut-off frequency.

#### 2.1.6. Gas Injection

Another active nucleate boiling enhancement technique that has been employed is the inclusion of dissolved inert gas in the working thermal fluids. Employing this enhancement technique, Kandlikar investigated the impact of the inclusion of dissolved inert gas in the FC-72 coolant. The researcher concluded that the dissolved inert gas provoked an appreciable reduction in the incipience temperature caused by the partial filling of the cavities of the heating surface with gas embryos. Nonetheless, because of the eventual removal of the dissolved gas from the surface cavities, the nucleate boiling heat transfer behavior at high heat fluxes was found to be like the one verified with the degassed FC-72 refrigerant. Accordingly, the researchers O’Connor et al. [54] and You et al. [55] arrived at similar conclusions and highlighted that the boiling incipience for the FC-72 refrigerant was sensitive to the dissolved inert gas at concentrations superior to 0.005 mol/mole. In the experimental work carried out by the authors Sarafraz et al. [56], the effects of SO_2_ gas injection on the water nucleate pool boiling heat transfer performance were studied. In this direction, the effects of different operating parameters, including the mole fractions of SO_2_ dissolved in water and different imposed heat fluxes up to 114 kW/m^2^, on the nucleate pool boiling HTC, nucleation site density, and bubble departure diameter were experimentally examined. The researchers found that the incorporation of SO_2_ in the vapor inside the bubbles, especially near the heat transfer surface, enhanced the nucleate pool boiling HTC. The investigation team also found that the nucleation site density could be described by an exponential function of the imposed heat flux and proposed a new prediction correlation for the nucleation site density that was able to estimate the mean bubble diameters and the local disturbance caused by the bubble frequency rate. In view of the experimental results, the authors stated that the HTC considerably increased with increasing gas rates dissolved in the water because of the higher mass transfer driving force between the SO_2_ captured inside the bubbles and the bulk of the solution and due to the increased local disturbance provoked by the gas injection. The researchers also concluded that the number of active nucleation sites, bubble nucleation, and diameter appreciably increased with an increasing SO_2_ injection rate. Nonetheless, the bubbles may have been generated due to the injection process and local disturbance due to the interaction of SO_2_ bubbles.

#### 2.1.7. Suction

The suction of the operating fluid in a nucleate pool boiling system can considerably improve the heat transfer behavior of the system. The improvement in the nucleate boiling heat transfer performance that can be accomplished through the active suction enhancement technique can be attributed to the enhancement of the fluid supplement and the increased bubble departure velocity caused by the local low pressure and shear lift force induced by the suction process. It should be stated that, for most active boiling heat transfer enhancement techniques, the detachment of the coalesced bubbles from the heating surface and the flow status of the working fluid have a great influence on the heat transfer behavior. On the one hand, when the fluid flow velocity increases, the thickness of the heat transfer boundary layer may be reduced, causing a delay in the onset of nucleate boiling. On the other hand, the heating surface will be covered by a vapor film at high heat fluxes, which will make liquid replenishing difficult, and the heat transfer performance will therefore deteriorate. Hence, the suction of the fluid and the bubbles perpendicular to the heating surface is one of the suitable active methods to enhance the pool boiling heat transfer performance. The method has therefore attracted more attention from the research community to fully understand its underlying mechanisms. A representative published work is the study carried out by the authors Zhang et al. [57], in which a smooth silicon chip was immersed in the subcooled FC-72 coolant to infer the nucleate pool boiling heat transfer behavior. The authors developed an apparatus incorporating a suction tube with different inner diameters of 2.2, 5.5, and 9.6 mm and tested the distances from the tube inlet to the chip heating surface of 1, 3, and 5 mm. For comparison purposes, the researchers also performed an experiment without suction on the same heating surface. In view of the obtained results, the authors deduced the following conclusions: (i) The suction pool boiling considerably improved the pool boiling heat transfer performance when compared to the result achieved without the suction process. The suction process enhanced the shear lift force of the vapor bubbles on the surface, increased the turbulent kinetic energy of the bubbles departing from the heating surface, increased the velocity of the two-phase motion, and facilitated fluid replenishing. (ii) The various distances between the suction tube inlet and the heating surface and the distinct tube diameters impacted the nucleate pool boiling heat transfer performance. For the same distance between the suction tube inlet and the heating surface, there was an ideal inner tube diameter that provided the highest CHF value and also possessed the highest HTC at high heat fluxes. For the same tube diameter, a shorter distance between the suction tube inlet and the heating surface promoted better heat transfer capability. Hence, the suction distance order for improving the heat transfer was 1 mm > 3 mm > 5 mm, and the suction tube inner diameter order for improving the heat transfer was 5.5 mm > 9.6 mm > 2.2 mm. (iii) The pool boiling process with suction presented higher CHF and HTC values than those obtained for pool boiling without suction. On the one hand, considering a tube diameter of 5.5 mm and 1 mm distance between the suction tube inlet and the chip heat transfer surface, the CHF increased by nearly 39.2% when compared to the value achieved with pool boiling without the suction process, having a maximum value of 33.4 W·cm^−2^. On the other hand, the HTC was enhanced by around 79.8% compared to that with the pool boiling process without suction, achieving a value of 1.093 W·cm^−2^·K^−1^.

#### 2.1.8. Local Jet Impingement

**Figure 8:**
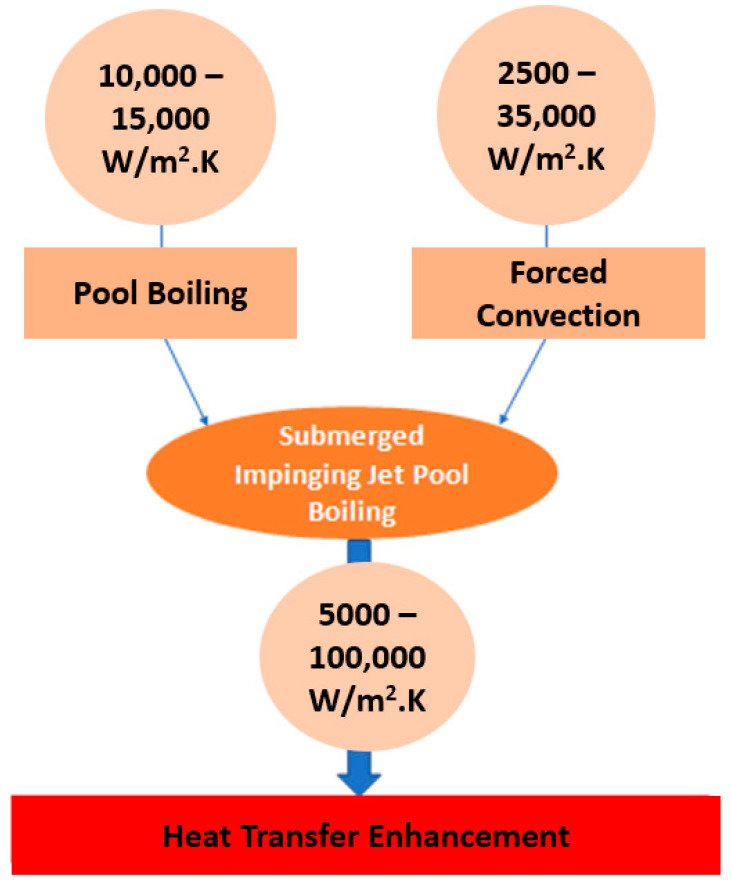
Schematic diagram showing the typical ranges of the heat transfer coefficients of the mechanisms involved in submerged impinging jet pool boiling. Adapted from [60].

The combined usage of boiling and jet impingement procedures is a relevant contributor to the development of effective and compact thermal management systems that deal with high heat flux dissipation requirements in applications such as computers, power electronics, and gas turbines. The impact of the geometric and working parameters on the single-phase jet impingement process that facilitates the achievement of the optimal pressure drop, flow rate, temperature uniformity, and heat dissipation capability is already known. The phase change heat transfer, together with the very high HTC achieved with the single-phase jet impingement, can enhance the heat removal capability and, at the same time, improve the temperature uniformity. Additionally, the sweeping of vapor from the heating surface through the action of the liquid jet greatly increases the CHF, especially when compared to that obtained with pure pool boiling [58]. Moreover, the researchers Ma and Bergles [59] investigated nucleate boiling using jet impingement for R113 with varying velocities, subcooling degrees, flow directions, and surface conditions. The authors employed jet diameters of 1.07 and 1.81 mm and two heaters of 5 × 5 mm^2^ and 3 × 3 mm^2^ dimensions. The authors confirmed that the temperature overshoot decreased with increasing jet velocity, which was attributed to the increase in the HTC with increasing velocity. Additionally, the surface status was also found to appreciably affect the fully developed boiling regime, with the boiling curves shifting because of the surface aging effect. Additionally, the authors Wolf et al. [60] studied the local free-surface planar water jet impingement boiling heat transfer characteristics with the aim of revealing the major factors impacting boiling heat transfer. The authors concluded that the heat transfer in the fully developed boiling regime was not influenced by the velocity of the jet but was instead determined by the evaporation and vigorous mixing provoked by the detachment of the bubbles. Nonetheless, in the single-phase and partial boiling regimes, the velocity of the jet had an appreciable impact on the HTC because of the convective heat transfer present in these regimes. The streamwise distance from the stagnation point showed a relative influence on the single-phase HTC but had no impact on the fully developed regime. Also, to ameliorate the thermal performance of the jet impingement process, the incorporation of enhanced nanostructures on the impinged surface has already been proposed [61]. Generally, the following concluding points concerning jet impingement heat transfer enhancement techniques can be listed: (i) In fully developed boiling regimes, the boiling curves for free circular or plan configurations are independent of the velocity and diameter of the jet and subcooling degree. Therefore, it can be inferred that the jet impingement boiling curve can be extrapolated from the pool boiling correlations. Nonetheless, the overshot temperature and CHF are influenced by the referred parameters. (ii) Conflicting findings and results have been published regarding submerged and confined jet impingement configurations in fully developed boiling regimes. Some researchers have found that the velocity of the jet and the degree of subcooling does not affect the boiling curve, whereas other authors have stated that these parameters strongly affect the nucleate pool boiling heat transfer behavior. Nonetheless, it can be argued that in the submerged configuration, the variation of the far-field temperature in the operating fluid greatly affects the effectiveness of the nucleate boiling heat transfer process, while in the confined configuration, the mixing of the vapor bubbles and the impinging jet is strongly promoted. (iii) The roughness of the heating surface has been found to be critical to jet impingement boiling heat transfer. (iv) The jet impingement boiling heat transfer has been found to be independent of the wettability (contact angle characterization) of the boiling surface in the single-phase convection, while it is affected by the mentioned wettability in boiling heat transfer. (v) In cases where the area of the heating surface is larger than the jet diameter and the thermal boundary condition is close to the wall heat flux, the existence of the single-phase convection at the stagnation zone and boiling at the far-field on the heating surface is likely. The diagram in Figure 8 shows the typical range of the heat transfer coefficients associated with the mechanisms involved in local jet impingement pool boiling heat transfer enhancement.

#### 2.1.9. Wall Deformation

It has already been found that one possible way to relieve the high temperature needs required for the onset of the nucleate boiling process is to increase the operating fluid stability by temporarily decreasing the pressure of the fluid. Accordingly, under confinement conditions, the low-amplitude dynamic deformation of the wall may change the liquid pressure, and hence, the morphing of the wall appears to be a promising attempt to control the onset of nucleate boiling and to reach boiling incipience at lower temperatures. The innovative active heat transfer enhancement technique that can be designated by wall deformation was proposed in the work performed by the authors Leal et al. [62]. The aim of the technique is to decrease the pool boiling incipience point through the dynamic deformation of the confinement wall in a narrow horizontal space. Such dynamic deformation generates pressure alterations that increases the fluid’s metastability. It was developed as an in-house-built experimental apparatus to measure the boiling incipience temperature, and the obtained results were compared with the those offered by the available theoretical hydrodynamic/nucleation models. The main experimental parameters were 625 µm maximum distance between the heat transfer aluminum cylinder and the confinement plate, a maximum deformation amplitude of 210 µm, and a frequency varying between 0 and 100 Hz, which were employed for determining the impact of frequency on the nucleate boiling incipience. The experimental results revealed a nearly linear decrease in the boiling incipience overheating with increasing frequency, which was interpreted by the authors as being the result of the liquid pressure variation. Also, the researchers noted that the dynamic deformation frequency increase increased the fluid pressure variation over time. During the liquid pressure reduction process, the overheating of the operating fluid increased due to reduction in the saturation temperature, and this increase may be enough for the onset of nucleate boiling. Additionally, for a better understanding of the impact of the deformation of the wall on boiling incipience superheating, the authors also proposed two different models: one hydrodynamic model and one nucleation model. In this sense, when using the nucleation model, the investigation team considered the pool boiling incipience as being independent of the level of confinement of the boiling heat transfer system. Finally, the boiling incipience temperature decreased when the deformation frequency of the wall was raised. Also, when wall deformation did not occur, the pool boiling incipience temperatures were consistent with the those given by the nucleation model. However, in cases where wall deformation was employed, the theoretical and experimental results differed considerably. For instance, within the range of frequencies considered, the decrease in the wall superheating was around only 2 K according to the nucleation model, while it was experimentally measured as being near 20 K. These discrepancies may reveal that the incipience temperature reduction was not provoked only by the reduction in fluid pressure. Indeed, other mechanisms should be considered to clarify the origin of these differences, like the transient and convection phenomena, and further experimental and numerical studies are required to better understand the decrease in the incipience point. The fundamental conclusion of the referred work was that the pool boiling incipience temperatures were reduced by the dynamic deformation of the wall effect. It also should be emphasized that for a deformation amplitude of 210 µm, a confinement distance of 625 µm, and a frequency of 100 Hz, the wall superheating at boiling incipience almost disappeared.

### 2.2. Passive Techniques

#### 2.2.1. Surface Modification

The surface coatings provide an efficient solution to modify the boiling surface according to the requirements of the heat transfer enhancement routes. Researchers have used different coating techniques with nucleate boiling heat transfer enhancement purposes, like sintering [63] and spraying [64], and have used metals, metal oxides, ceramics, polymers, and composites to obtain heating surface morphologies that incorporate porous nanostructures, pyramids, pin fins, tunnels, and pillars. This kind of modification greatly affects the diverse practical features of the nucleate pool boiling heat transfer behavior, including the number of active nucleation sites, surface roughness, wettability, and porosity. The characteristics of the surface, temperature, and operating pressure, together with the heat transfer fluid’s thermophysical properties, are the main factors for adjusting the nucleate boiling heat transfer performance. From the point of view of nucleate boiling heat transfer enhancement, the most relevant thermophysical properties of the heat transfer fluid are the thermal conductivity, specific heat, viscosity, density, and surface tension, and the most prominent properties of the surface are its morphology, wettability, and roughness. The modified enhanced surfaces for boiling heat transfer improvement facilitate nucleation and maximize the CHF value by delaying the transition to the film boiling regime. For instance, the cavities existing in the heating surface nucleate bubbles and entrap them inside the cavities, and the combined usage of the surface roughness and the cavities augment the number of active nucleation sites during the nucleate boiling regime. The surface modification techniques for heat transfer enhancement can be mechanical processes, like sandblasting and laser machining [65]; surface coating processes, like chemical vapor deposition (CVD), physical vapor deposition, spraying, and plasma; chemical processes, like oxidation and etching [66]; and microelectromechanical systems (MEMS) [67]. In the literature of interest, the performance of any modified coated heating surface is evaluated by comparing its nucleate HTC and CHF values with those provided by an untreated surface.

##### Nanoparticles

**Figure 9:**
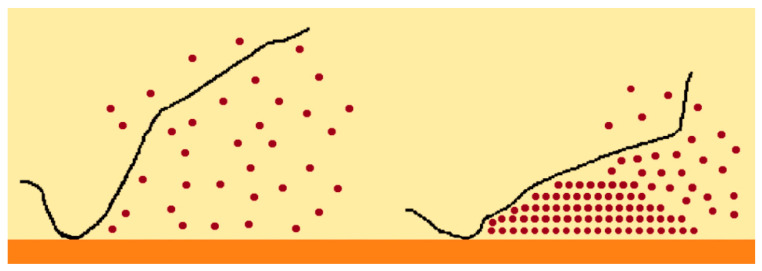
Schematic diagram of nanoparticle deposition in microlayer evaporation during boiling.

The deposition of nanoparticles on the heat transfer surface during the boiling process, which is illustrated in the schematic diagram in Figure 9, may form porous coatings that increase the heat transfer area and wettability of the surface and decrease its contact angle. Hence, an increase in the wettability and heat transfer area causes an appreciable increase in the nucleate boiling CHF value. The authors Watanabe et al. [68] investigated the effects of the adhesion of a nanoparticle coating and its eventual detachment on the nucleate boiling heat transfer capability enhancement. The researchers highlighted that the adhesion of the nanoparticle coating depended on the nature, dimensions, and distribution of the nanoparticles. Both the HTC and CHF values increased with the nanoparticle coating. Moreover, the authors Wu et al. [69] used titania and silica nanoparticles with 10 µm diameter to spin-coat a copper surface and form a hydrophilic boiling enhanced surface to infer the HTC and CHF of water and FC-72. The experimental results showed that the hydrophilicity of the enhanced surface achieved considerable enhancements in the HTC and CHF values compared to those obtained with the uncoated boiling surface. Also, the authors Forrest et al. [70] employed the layer-by-layer deposition method to assemble multiple layers of silica nanoparticles onto a nickel wire and a stainless steel plate, aiming to increase the boiling heat transfer performance. The assembly involved the deposition of a bilayer in which the substrate was first immersed in a positively charged solution, forming a layer of positively charged species, and then in a negatively charged solution, forming, in turn, a layer of negatively charged species in sequence. Using suspensions of 24 µm diameter silica nanoparticles, bilayers were formed, obtaining hydrophilic, superhydrophilic, and hydrophobic surfaces. The superhydrophilic ones also involved calcination at 550 °C for four hours in a furnace to make the coating porous, which increased the heat transfer area. The hydrophobic surfaces were produced by immersing the calcinated surfaces in fluorosilane and then placing them in an oven at 140 °C. The experimental results indicated that the nucleate HTC significantly increased, and the CHF value increased with the increasing thickness of the superhydrophilic coating caused by the higher number of nanoporous structures. Apart from being assembled onto surfaces, the nanoparticles can act as masking materials in conjunction with diverse etching methods to produce certain features on the modified surfaces. For instance, the authors Chen et al. [71] used a self-masking technique with nanoparticles as etching masks released from a dummy material, for example, a cover glass, during the etching procedure to form high-aspect-ratio nanopillars of polymeric materials, like polydimethylsiloxane (PDMS). Additionally, the authors White et al. [72] employed the electrophoretic deposition technique to coat a stainless steel plate. The nanoparticles were used with other coatings to form a hybrid coating due to their properties, which enabled them to augment the nucleation sites for bubble generation and increase the heat transfer area. In addition, the researchers Yeom et al. [73] used 4 to 6 µm sized nanoparticle coatings produced by the electrophoretic deposition of 60 to 80 nm sized titanium and titania nanoparticles. It was found that the coatings increased the bubble departure frequency but provoked a decrease in the bubble departure volume and nucleation site density. Though the coatings improved the water CHF value, they also delayed the boiling incipience. Overall, the titania coating provided a greater CHF enhancement than the titanium coating, but the latter one was found to be more robust. Nanoparticle deposition on a substrate can also be achieved by nanofluid boiling, which is controlled by the boiling duration, concentration of nanoparticles, and imposed heat flux [74,75]. Also, the researchers Kiyomura et al. [76] evaluated the water boiling behavior using coated copper heating surfaces with thickness ranging from 0.05 to 0.23 µm, achieved by the deposition of iron oxide nanoparticles with a mass fraction ranging from 0.029 to 0.29 g/L. The highest nucleate HTC value was obtained using the smoothest heating surface with the deposition of nanoparticles with a low mass fraction. All the other tested surfaces deteriorated the heat transfer behavior because of the enhanced thermal resistance and decreased the nucleation site density stimulated by the thicker layers. Moreover, the researchers Souza et al. [77] found that the deposition of 10 nm sized nanoparticles during pool boiling improved the HTC when using HFE-7100 as the heat transfer fluid, while the 80 nm sized nanoparticles decreased the HTC when compared to the value obtained without deposition of the nanoparticles on the heating surface. The researchers also highlighted the weak reproducibility of the nanoparticle layer formed initially by nanofluid boiling, pronounced by the formation of random multiscale active nucleation sites. Generally, it seems that the nanoparticle deposition mitigated some of the limitations entailed by the general use of nanofluids, like sedimentation, agglomeration, precipitation, and surface degradation by erosion, which may induce fluctuations in the heat transfer performance with time. Nonetheless, further in-depth studies are required to better understand the strength, durability, and homogeneity of the nanoparticle deposition during the boiling process.

##### Nanotubes

**Figure 10:**
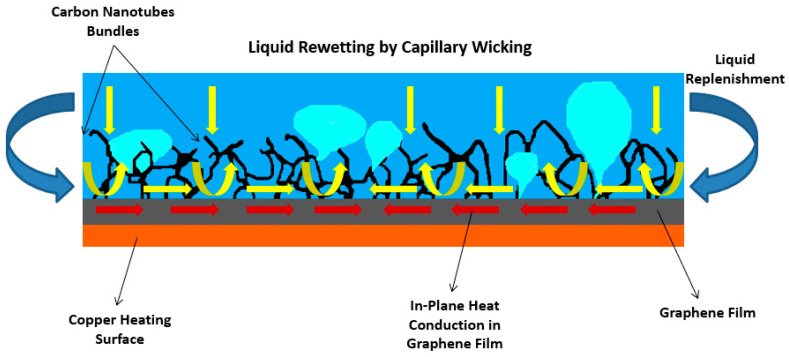
Schematic representation of the liquid replenishment process through the capillary wicking effect of CNT bundles. Adapted from [82].

Carbon nanotubes (CNTs) are ultra-thin tubes of graphitic carbon with typical outer diameters ranging between 1 and 100 nm and lengths from 1 to 50 µm. CNTs have been chosen as coating materials for nucleate boiling enhancement purposes due to their high thermal conductivity and superior mechanical properties. The synthesis of CNTs relies fundamentally on techniques like electric arc discharge, laser ablation, and the different alternatives of chemical vapor deposition (CVD) [78]. Other fabrication techniques include flame synthesis, electrolysis of molten halide salts, and cracking of hydrocarbons. Among all the production techniques, plasma-enhanced chemical vapor deposition (PECVD) is the most suitable approach to form aligned, individually standing, and size-controlled CNTs. Furthermore, the wettability of individual CNTs has been extensively investigated, and the fundamental conclusion is that materials with relatively low surface tension can wet the CNTs’ outer surface with contact angles lower than 90°. Also, a forest of vertically aligned CNT grown and deposited on a PAN-based carbon fiber formed hierarchical surfaces at the micro- and macroscales, in which the CNTs induced a superhydrophobic characteristic. Moreover, the authors Ahn et al. [79] produced multiwall carbon nanotubes (MWCNTs) by CVD on silicon surfaces to generate 9 and 25 µm tall CNTs in vertically aligned forests to evaluate their nucleate pool boiling heat transfer performance. The MWCNTs had a diameter between 8 and 16 µm and a pitch between 8 and 16 nm. The heat transfer pool boiling performance achieved the same magnitude level for both heights of the nanotubes, but the taller nanotubes resulted in a 28% enhancement in the CHF value when compared to only a 25% enhancement using the shorter nanotubes. This fact was interpreted based on the fact that the taller nanotubes provided more suitable pathways for the liquid to flow to the nucleation sites. The authors also explained the heat transfer enhancement of the silicon surfaces modified with CNTs during the film boiling regime, compared to the bare silicon surfaces, based on factors that included the CNTs’ enhanced thermal conductivity, larger cold spots, enhanced ability to collapse the vapor film, improved liquid–solid contact area, and increased heat transfer area. In the case of CNT coatings on a metal substrate, the CNTs act as hydrophobic dots, augmenting the active nucleation site density and the bubbles generated over the CNT coatings, while the untreated metal surface in their surroundings works as a hydrophilic region, delaying the vapor film formation. Moreover, the authors Ujereh et al. [80] examined the nucleate boiling heat transfer behavior of silicon and copper boiling surfaces coated with arrays of CNTs with different densities and area coverages by the plasma-enhanced chemical vapor deposition (PECVD) technical solution. The researchers found that the silicon surface coated with light CNT arrays was more capable of reducing the incipience superheat and increasing the FC-72 nucleate HTC and CHF. Such evidence was interpreted by the authors as being the result of the ability of the CNT mesh to offer a higher number of zero cone angles and cavities that were ripped for nucleation with minimum superheat. Also, the researchers reported that increasing the CNT mesh density on the silicon surface provoked only a minor decrease in the incipience superheat. Nonetheless, very dense CNT arrays decreased the CHF by decreasing the available heat transfer area when compared to the effect of lighter CNT arrays. Additionally, a greater nucleate boiling heat transfer enhancement was obtained with CNTs incorporated on silicon heating surfaces than on copper ones because the uncoated copper surfaces were rougher than the uncoated silicon surfaces, which offered abundant active nucleation sites. Additionally, graphene has received much attention from researchers studying nucleate boiling heat transfer applications because of its properties. In this sense, the authors Jaikumar et al. [81] examined the nucleate boiling heat transfer behavior of graphene and graphene oxide coatings applied onto a copper heating surface by dip coating and reported increased performance with increasing thickness of the coatings, staying a 47% and 42% enhancement in the HTC and CHF, respectively. Moreover, the researchers Kumar et al. [82] investigated the nucleate boiling heat transfer performance of hybrid CNTs together with graphene nanostructures. To this end, the authors grew many heterostructures of graphene/CNTs on a copper heating substrate through the PECVD technique. The authors confirmed a 155% enhancement in the HTC, a 40% increase in the CHF, and a reduction of 62% in the boiling superheat. Figure 10 shows the CHF enhancement mechanisms, such as liquid replenishment to the heating surface through capillary wicking that is induced by the CNT bundles. This effect delays the CHF occurrence.

The titanium oxide nanotubes have also been used by many researchers to properly modify heat transfer surfaces. In this direction, the authors Wang et al. [83] produced titanium oxide nanotube surface arrays by electrochemical anodization of a plain titanium surface. The authors found that the wettability of the surface could be adjusted through the application of ultra-violet irradiation, defining the photo-induced hydrophilic (PIH) effect. The titania nanotube-composed layer had a superhydrophilic nature in cases where it was illuminated with ultra-violet light and gradually returned to its superhydrophobic character when it was irradiated with visible light. The wettability change was also verified in the case where the titania nanotubes surface was modified with self-assembled monolayers of certain silanes and acids. This technique diminished the water nucleate HTC value but increased the CHF of the water. Finally, the authors Chen et al. [84] achieved an enhancement in the water nucleate HTC using a titanium surface coated with titanium oxide nanotube arrays (TNTAs) compared to that achieved with a bare, plain titanium surface. The experimentally coated surfaces demonstrated a contact angle close to zero, whereas the bare, plain titanium heating surface exhibited a contact angle of around 70°. Also, the boiling surfaces modified with TNTAs yielded nearly half the value of the wall superheat at any given heat flux compared to the wall superheat of a bare titanium surface.

##### Nanowires and Nanofibers

Nanowires are nanoscaled rods with up to tens of nanometers of diameter and a high length-to-diameter ratio. Silicon has already shown promising electrochemical properties when used in electroless metal-particle-assisted etching in silicon nanowire production. The etching process may involve, for instance, the immersion of a silicon wafer into an aqueous solution of silver nitrate and hydrofluoric acid as etchant. Moreover, the authors Yao et al. [85] formed copper nanowires on silicon surfaces without any interfacial bonding layer and controlled various parameters to define the height of the nanowires. The authors reported that the adopted method of synthesis gave extra longevity and adhesion interfacial strength to the copper nanowires. The researchers also deduced that the absence of an epoxy layer between the nanowires and the surface reduced the existing interfacial thermal resistance. A gradual enhancement in the HTC for water was obtained with increasing nanowire heights from 2 to 35 µm, which was attributed to the increased size and density of cavities. Additionally, the researchers Kumar et al. [86] investigated the effects of the copper nanowire diameter, ranging between 35 and 200 nm, on the FC-72 nucleate boiling heat transfer performance and revealed that the HTC and CHF values increased with the increasing diameter of the nanowires. Such evidence was explained by the increase in the cavity size and density, which were obtained by the coagulation and grouping of the nanowires during the drying stage of the employed template-based electrodeposition. Apart from that, the authors Wen et al. [87] manufactured a hierarchical surface composed of long copper nanowire arrays surrounded by short copper nanowires, whose specific arrangement induced the formation of microcavities between the clusters of nanowires. The authors reported an enhancement in the water nucleate pool boiling behavior, which was attributed mainly to the increased density of nucleation sites, fluid rewetting by capillary wicking, and separation of the liquid and vapor pathways. The modified surface achieved a 37% lower incipience superheat, a 185% higher nucleate HTC, and a 71% higher CHF compared to those obtained using a plain copper surface. Furthermore, the researchers Ray et al. [88] synthesized titanium oxide nanowires normally oriented against a copper heating surface and with thickness ranging between 150 and 450 nm by the glancing angle deposition technique with the aid of an electron beam evaporator. The researchers confirmed an enhancement of the R-134a coolant nucleate boiling thermal performance of the titania nanowire arrays compared to using a plain boiling surface. The peak performance was achieved by employing a 45 nm thick coating, which yielded a nearly 44.9% decrease in the incipience superheat and around 81.3% enhancement in the nucleate HTC value. Apart from that, it can be stated that nanofibers are fibers fabricated from polymers with typical diameters of less than 100 nm. Additionally, the authors Jun et al. [89] evaluated the nucleate boiling heat transfer behavior of copper-plated electrospun copper nanofibers and found out that the water and ethanol HTCs were three to eight times higher than those achieved using a plain bare surface made of copper, but the authors did not report any considerable enhancement in the CHF value. The nucleate boiling heat transfer enhancement was explained by the researchers as being the result of the ability of the technique to increase the temperature of the bubbles surrounding the working thermal fluid, thereby assisting more effective bubble growth.

#### 2.2.2. Surface Modification by Materials and Structures

##### Porous Coatings and Structures

**Figure 11:**
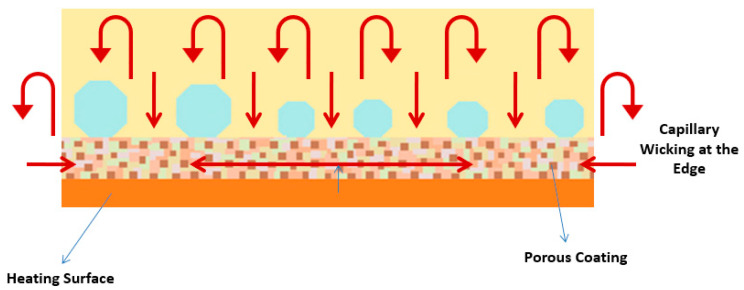
Schematic representation of the capillary wicking effect on a porous coating. Adapted from [91].

**Figure 12:**
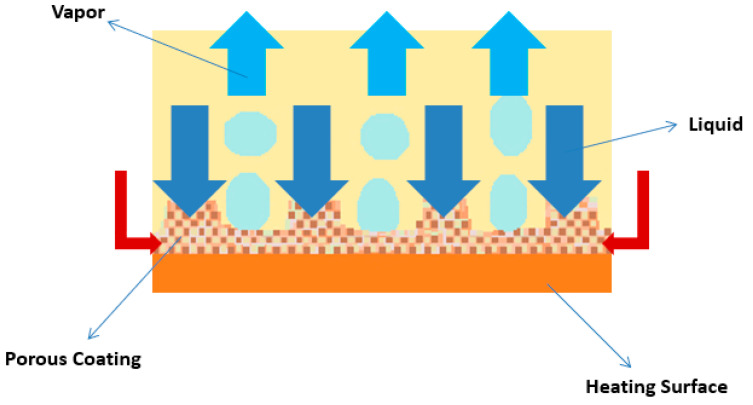
Schematic representation of the separation of the liquid and vapor paths on a porous coating. Adapted from [91].

The microporous coatings prepared for nucleate pool boiling heat transfer enhancement purposes have intrinsic characteristics, like increased available heat transfer area, enhanced density of active nucleation sites, capillary wicking effect [90], and segregation of the liquid and vapor pathways, which greatly improve the nucleate pool boiling performance. The capillary wicking and liquid–vapor path separation are schematically described in Figure 11 and Figure 12, respectively.

The microporous coatings can be produced by a wide variety of techniques, like welding, sintering, electrolytic deposition, and flame and polymer plasma spraying [92]. The goal of the techniques is the synthesis of a layer full of potential nucleation cavities. Moreover, the researchers Jun et al. [93] assessed the water boiling heat transfer performance employing microporous surfaces fabricated by brazing copper particle coatings with an average size of 25 µm and thickness between 49 and 283 µm. Indeed, the authors identified three different heat transfer regimes: (i) microporous regime, in which the HTC and CHF values increased with increasing coating thickness; (ii) microporous-to-porous transition state, in which the CHF increased with increasing coating thickness, whereas the HTC increased at lower heat fluxes and decreased at higher heat fluxes; and (iii) porous regime, in which the HTC and CHF decreased with increasing coating thickness. Additionally, the authors Seo et al. [94] compared the FC-72 CHF using the following hydrophilic surfaces: (i) non-porous graphene layer deposited surface; (ii) non-porous SiC layer deposited surface; (iii) porous graphene layer deposited surface; and (iv) porous SiC layer deposited surface. The substrate was a plain indium tin oxide surface, and compared to its CHF value, the CHF values for the different tested surfaces were 9%, 16%, 90%, and 58% higher, respectively. The different magnitude values were interpreted by the authors based on the heat dissipation limits determined by the thermophysical properties of graphene and SiC and on the hydrodynamic and capillarity effects provided by the morphology of the surface and its porous structure. In the study conducted by the researchers Sarangi et al. [95], it was confirmed that irregular-shaped particles yielded a higher FC-72 nucleate HTC than that obtained using spherical particles with uniform coating porosity. The authors also proposed that the nucleate HTC and CHF were profoundly influenced by the porosity fraction of the coating, pore diameter, interfacial area, thermal conductivity, and permeability. Additionally, the researchers Nishikawa and Ito [96] proposed Equation (1) for the nucleate boiling regime for porous modified boiling surfaces:

(1) $$\frac{q^{\prime \prime }\delta }{{∆T}_{sat}K_{m}}=0.001{\left(\frac{\sigma^{2}h_{fg}}{q^{\prime \prime 2}\delta^{2}}\right)}^{0.0284}\cdot {\left(\frac{\delta }{q_{p}}\right)}^{0.56}\cdot {\left(\frac{q^{\prime \prime }d_{p}}{\varepsilon h_{fg}\mu_{g}}\right)}^{0.593}\cdot {\left(\frac{K_{f}}{K_{m}}\right)}^{0.708}\cdot {\left(\frac{\rho_{f}}{\rho_{g}}\right)}^{1.67}$$

where δ, d_p_, and ε are the thickness of the coating, diameter of the particle, and porosity fraction of the coating, respectively. The thermal conductivity of the coating K_m_ can be determined as a function of the fluid thermal conductivity K_f_, particle thermal conductivity K_p_, and porosity, given by Equation (2):

(2) $$K_{m}=K_{f}+\left(1-\varepsilon \right)K_{p}$$

Assuming that each pore contains a vapor bubble surrounded by spherical particles and acts as an active nucleation site, the researchers O’Neill et al. [97] proposed a heat transfer prediction model for porous surfaces and different working fluids based on Equation (3):

(3) $${∆T}_{sat}=\frac{\beta q^{\prime \prime }r^{2}}{K_{f}}-\frac{2\sigma }{\left(dP/dT\right)r}$$

where β is the type of packing and density of active nucleation sites factor, and r is the pore radius. Additionally, the researchers Jaikumar et al. [98] and Protich et al. [99] examined the boiling heat transfer capability enhancement using surface microstructures containing graphene oxide and 15 µm sized copper particles. The microstructures were produced by screen printing, chronoamperometry electrodeposition, and galvanostatic electrodeposition technological solutions. The water nucleate HTC enhancement was attributed by the authors to the increased number of nucleation sites and formation of wicking dendritic structures. The CHF value also suffered a considerable increase caused by the improved wettability derived from the combined effect of increased surface roughness and the formation of dendritic structures. Additionally, the researchers Li and Peterson [100] found that the boiling heat transfer performance was dependent on certain factors inherent to the porous coatings, like the coating thickness, porosity, and mesh size. The authors proposed that the heat from the substrate was initially conducted through the skeleton of the porous structure, then transferred to the fluid through convection, and finally dissipated on the porous surface by boiling or evaporation. This sequence revealed that a porous coating with enhanced thermal conductivity could improve the boiling heat transfer capability, particularly at low imposed heat fluxes. The same authors also proposed various heat transfer enhancement mechanisms, including increased wetted area, increased active nucleation site density, greater interaction among bubbles, the film evaporation effect, and capillary wicking within the porous structure. Because of their facile preparation method, the nanoporous membranes of various materials produced by chemical or electrochemical technological solutions have proved to be very suitable for heat transfer purposes. For instance, silicon porous structures are nowadays commonly employed, and single-crystalline silicon can be obtained electrochemically by galvanostatic anodization using hydrofluoric acid solutions. The silicon atoms are dissolved in the nanoscale pores formed in the bulk, and the single-crystalline silicon is then converted into an anodized porous film. Additionally, the researchers Tang et al. [101] and Lu et al. [102] fabricated nanoporous copper surfaces with pores with diameters ranging from 50 to 200 nm by the hot-dip galvanizing/dealloying (HDGD) process. The authors achieved a 63.3% reduction in the incipience superheat and a 172.7% enhancement in the water nucleate HTC value. However,, the nanostructure and porosity were observed to change only slightly after 100 h of the boiling process, and the nanostructure continuously coarsened over time. Furthermore, the authors Hendricks et al. [103] manufactured flower-like zinc oxide nanostructures on aluminum and copper substrates by the low-temperature micro-reactor-assisted deposition approach. The authors found reductions ranging between 25 and 38 °C in the wall superheat during the water nucleate boiling regime and up to a 4-fold increase in the CHF value. Generally, the nanoporous structures used bring with them diverse challenges concerning their use under pool boiling scenarios. One such challenge is the surface nanostructure longevity and considering the changes in the heat transfer performance in the already performed extended lasting tests, it is vital to address the any changes in the heat transfer surfaces over time in the nanostructure of the surfaces. Moreover, it is possible to simultaneously form nanostructures like rods, fiber arrays, and porous structures using templates with nanopores. The nanopores of the templates are uniform and dense, providing a very promising tool to produce a high yield of nanostructures, for instance, nanorod arrays [104]. A subsequent removal of the templates produces a porous network that replicates the template.

##### Tunnels and Reentrant Cavities

**Figure 13:**
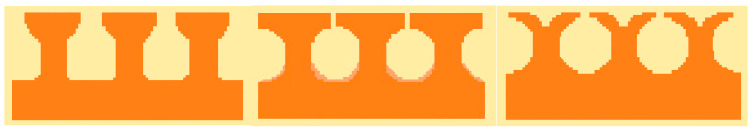
Typical cross sections of reentrant cavities used in pool boiling. Adapted from [111].

The authors Nakayama et al. [105] devised a boiling enhancement scheme that consisted of first gouging the heating surface with 0.25 mm wide and 0.4 mm deep parallel rectangular tunnels and then covering it with a thin copper plate with rows of 50 to 150 µm sized pores. By applying such a technique, the R-11 coolant boiling curve exhibited a tendency mainly dictated by the density and diameter of the pores. The same authors proposed the following boiling modes with this heat transfer enhancement surface: (i) dried-up mode, where the tunnels are filled with vapor and the boiling is conducted outside the tunnels through a mode similar to that provided by a plain heating surface with regularly spaced active nucleation sites; (ii) suction–evaporation mode, in which the departure of the bubbles from the active pores causes the fluid to be sucked by the inactive pores into the tunnels, from which the liquid spreads and evaporates, offering the best boiling heat transfer performance of all boiling modes; and (iii) flooded mode, where most of the tunnels are flooded by the working thermal fluid and one active pore acts as a single nucleation site. Moreover, the authors Pastuszko [106] conducted experimental work and a theoretical analysis of the nucleate boiling heat transfer capability using surfaces with interconnected narrow horizontal and vertical tunnels, designated as narrow tunnel structures (NTSs). In addition, water, ethanol, and the R-123 commercial refrigerant were employed as the heat transfer fluids for the boiling process at atmospheric pressure. The tunnels had external covers manufactured from 0.1 mm thick perforated copper foil with hole diameters ranging from 0.3 to 0.5 mm, sintered with the mini-fins formed on the vertical side of 10 mm high rectangular fins and the horizontal inter-fin surface. The tunnel surfaces allowed the separation of the fluid suction sites (pores in the vertical tunnels) from growing bubbles at the tunnel outlet. A large cross section of tunnel outlets ranging from 3 to 7.5 mm^2^ allowed intensive bubble generation at higher heat fluxes. Both the proposed prediction models and experiments confirmed that the use of NTSs improved the nucleate pool boiling heat transfer at atmospheric pressure. Additionally, the authors Halon et al. [107] conducted low-pressure water pool boiling using finned NTSs (NTS-1, NTS-2, and NTS-3) and tunnel structures (TS-1, TS-2, and TS-3). A nucleate HTC value that can be achieved under low-pressure conditions with a pressure between 0.75 and 4 kPa was estimated and compared with the HTC provided by a plain heating surface. The differences in the design of the surface reduced the wall superheat, thus allowing the mitigation of the impact of the sub-atmospheric conditions. The obtained experimental results indicated that the tunnels substantially improved the heat transfer at low pressures, even as high as by the order of magnitude, compared to a plain surface. In comparison to the boiling process occurring at atmospheric pressure, the boiling curves determined at low pressures were shifted towards higher superheats, and the heat transfer decreased. The introduction of enhanced TS or NTS surfaces allowed this negative effect to be reduced because of the extended heat transfer area and higher density of artificial nucleation sites due to the perforated copper foil. During the heat transfer boiling process, the bubbles were no longer detached from the foil perforations, which was caused by the fact that the nucleation site critical radius was larger due to alterations in the vapor density and surface tension, and consequently, the vapor was released through the horizontal endings of the tunnels instead of the perforations. At sub-atmospheric pressure, the TS structures’ HTC was superior to that of the NTS structures. The best NTS-3 structure was still at the level of the worst TS-1 structure. This result contradicted the outcome of experiments conducted at atmospheric pressure, like the one by Pastuszko et al. [106], who found that the best enhancing structure at the atmospheric pressure was the NTS with the narrowest tunnel. Here, the NTS-1 was found to be the worst NTS structure, yielding an HTC similar to that provided by a plain heating surface. Neither its increased heat transfer area nor narrower tunnels considerably influenced the nucleate HTC. The best heat transfer performance was achieved using the TS-2 surface with the thickest mini-fins covered with perforated foil. As a result, the thermal mass of the surface was the largest, the tunnels were smaller, and the fluid evaporated more quickly. The nucleation on the TS-2 led to a small superheat and an increased nucleate HTC value. Moreover, the researchers Moita et al. [108] emphasized the relevance of bubble dynamics and bubble interaction in the nucleate pool boiling heat transfer enhancement, noting that an improper control of such factors might lead to the generation of larger bubbles, with strong coalescence and vapor blanket formation. Also, the authors Teodori et al. [109] employed particle image velocimetry (PIV) measurements to identify an optimum array of cavities, which could counterbalance the beneficial feature of the activation of nucleation sites and the disadvantageous effect of the horizontal coalescence of the vapor bubbles. Furthermore, the authors Ji et al. [110] performed the R134a pool boiling process on a heating surface composed of tubes with reentrant cavities. The experimental results indicated that the ideal enhanced imposed heat flux region seemed to be within 40 kW/m^2^ when using the reentrant cavity-improved tubes. The heat transfer behavior was appreciably ameliorated at imposed heat fluxes lower than 200 kW/m^2^, and a substantial increase in the nucleate HTC value, up to 330% higher than that obtained with the plain tubes, was confirmed by the authors. Nevertheless, at imposed heat fluxes higher than 200 kW.m^2^, it was found that the nucleate HTC of the enhanced reentrant cavity tubes was lower than the HTC value reached with the plain tubes. Some typical reentrant cavities used in the pool boiling heat transfer enhancement process are depicted in Figure 13.

##### Wicking and Grooved Surfaces

Diverse published studies have demonstrated that the monolayer wick of the sintered copper nanoparticle heat transfer technique combined with wick tridimensional structures results in a water nucleate HTC value as high as 200,000 W/m^2^·K and an enhanced 6-fold CHF value compared to that provided by plain copper surface water pool boiling [112]. The authors Nasersharifi et al. [113] proposed diverse multilevel wicks of columnar, monolayer, and mushroom posts wicks for controlling the liquid and vapor phase change flow in the pool boiling conditions using n-pentane as the working fluid. The surface was fabricated using 200 µm copper particles by multistep sintering. The monolayer wicks with and without the mushroom post structure provided 87% and 20% CHF enhancements, respectively, compared to those obtained when using a plain copper surface. Although the behavior of the mushroom was like that provided by the columnar post wick at low heat fluxes, the mushroom wick significantly delayed the surface dry-out through the continuous liquid supply by means of the mushroom cap. The researchers noted that the CHF enhancement of the mushroom wick was attributed to the pitch of 3.5 mm. The further reduction in the pitch to 1 mm led to a very considerable 250% enhancement in the CHF value. The columnar and mushroom posts with monolayer induced a 10-fold increase in the nucleate HTC when compared to the HTC provided by a plain boiling surface because of the reduced thermally conductive pathway through the thin monolayer wick under the controlled vapor region using the columnar and mushroom post wick. The authors confirmed that the liquid-filled monolayer significantly reduced the superheat by decreasing the effective thermally conductive path, whereas the columnar posts modulated the hydrodynamic instability wavelength, causing the CHF value to increase. In the work performed by the researchers Raghupathi et al. [114], it was demonstrated that extending the contact line regions within the base of the nucleating bubbles led to CHF value enhancement. The creation of a liquid meniscus adjacent to the 10 to 20 µm deep microgrooves in the bubble base area was responsible for the extended contact line regions. The depth of the microgrooves was determined to be such that enough fluid was present in the meniscus to maintain the evaporation process in the contact line region throughout the bubble lifecycle. The water pool boiling was conducted on copper surfaces with 10 to 100 μm deep and 100 to 500 μm wide microgrooves executed by CNC machining. The authors found that with 10 μm deep shallow grooves, the CHF initially increased with increasing groove width, reached the maximum of 168 W/cm^2^ with a groove width of 300 μm, and decreased with further increasing groove widths. The increase in the CHF was accompanied by an increase in the nucleate HTC up to 109 kW/m^2^·K. The evaporation of the fluid retained in the meniscus as the bubble grows over the microgroove walls appeared to be the main reason for the enhanced CHF value, rather than the capillary wicking of the fluid. Also, when 100 μm deep grooves were employed, the CHF increased with increasing groove width, reaching a peak of 124 W/cm^2^, which was lower than that obtained using a plain boiling surface. The CHF variation over the micro-grooved surfaces confirmed that the different heat transfer capability driving mechanisms were affected by the groove depth. Finally, the study conducted by the authors Deghani-Ashkezari and Salimpour [115] evaluated the impact of the geometry of the heating surface grooves on the pool boiling of water and the impact of an aqueous titania nanofluid. To allow comparison of the experimental results, they were performed on a smooth copper heating surface with diverse grooves of square, semi-circular, and triangular shaped cross sections and a spacing of 1.5 mm between them through the wire-cutting machining technique. The heating transfer areas of the plain surface and the surfaces with square, semi-circular, and triangular grooves were around 1590, 3149, 2500, and 3542 mm^2^, respectively. In view of the experimental results, the researchers made the following concluding remarks: (i) The grooves on the heating surface increased the heat transfer capability of the system during the water and nanofluid boiling processes. (ii) Compared to a plain heating surface, the triangular and semi-circular grooved boiling surfaces exhibited greater nucleate HTC values. The square grooved surface exhibited the weakest thermal performance, and the nucleate HTC value was only enhanced at low superheat values. (iii) The triangular grooved heating surface presented the best nucleate pool boiling performance and enhanced the HTC value by 120%, nearly 84%, and around 66% for water and 0.2% and 0.4% vol. concentration nanofluids, respectively, whereas the values for semi-circular grooves were only around 51%, 41%, and 35%, respectively. (iv) The enhanced heat transfer induced by the grooved surfaces usage was not merely due to the increased heat transfer area but also due to the situation of the grooves. Moreover, the authors Tang et al. [116] studied the boiling heat transfer characteristics of multilayer micromesh copper surfaces and stated that these surfaces exhibited a considerable enhancement in the pool boiling heat transfer capability, including the critical heat flux, heat transfer coefficient, and onset of nucleate boiling, in comparison to those of a plain copper heating surface. The micropores formed by the multilayer micromeshes enhanced the heat transfer behavior via an extended surface area, increased the number of active nucleation sites, and ameliorated the capillary wicking effect. Also, the authors observed that the increase in the layers of micromeshes decreased the size of the micropores, increased the density of nucleation sites, and improved the capillary wicking ability, further increasing the HTC and delaying the critical heat flux. The highest results of the study were CHF of 207.5 W/cm^2^ and HTC of 15.6 W/(cm^2^·K).

##### Fins and Studs

The authors Mudawar and Anderson [117] provided a methodology for producing hybrid nucleate boiling heat transfer enhancement surfaces involving certain features as follows: (i) a single extended cylindrical stud; (ii) square microfins machined along the complete perimeter of the stud; and (iii) nanoscale cavity-inducing surface finish by vapor blasting. This multiscale heat transfer enhancement scheme was very effective in eliminating the incipient overshoot and improving the pool boiling thermal performance. Also, the FC-72 heat transfer behavior was particularly enhanced with 30 °C subcooling. Such a subcooling degree stimulated a highly reduced vapor bubble volume. These characteristics are very useful in the cooling of an array of vertically oriented surfaces, where any eventual effect of the decrease in vapor production from the base heating surface on the surfaces mounted above would be minimized. A comparison of the FC-72 saturated and subcooled nucleate boiling processes showed the absence of incipience excursion and exhibited CHF values as high as 105.4 W/cm^2^ for saturated boiling, which was equivalent to a 7.5-fold enhancement when compared to the CHF obtained with a plain boiling surface, and as high as 159.3 W/cm^2^ for subcooled boiling, which corresponded to a 11.3-fold enhancement when compared to the CHF obtained with a plain heating surface.

##### Fins and Porous Structures

**Figure 14:**
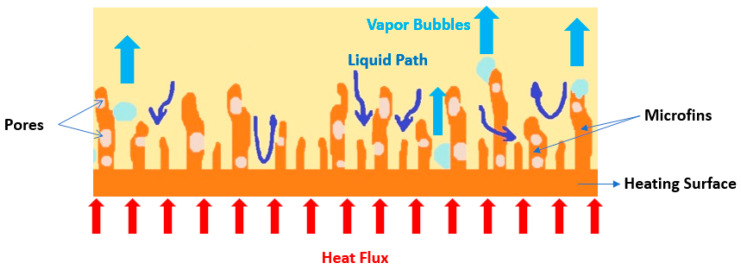
Schematic illustration of the liquid–vapor interactions on a surface with microfins and pores. Adapted from [121].

The authors Rainey and You [118] evaluated the nucleate boiling heat transfer performance using a hybrid enhanced surface with square microfins of 1 × 1 mm^2^, heights up to 8 mm, and a microporous coating. The researchers found an appreciable enhancement in the FC-72 refrigerant nucleate boiling heat transfer and CHF when compared to those achieved with a plain boiling surface. The authors also reported that the CHF enhancement was independent of the height of the fins for fin heights lower than 4 mm. They also argued that the nucleate boiling HTC and the CHF were affected by diverse conflicting factors, including the extended available heat transfer area, fin effectiveness, heating surface microstructure, vapor bubble detachment resistance, and resistance to the operating fluid rewetting. The investigation team also found moderately better heat transfer performance with a boiling surface horizontally oriented and with vertical fins than with a heating surface vertically oriented and with horizontal fins. Also, the authors Chang and You [119] studied the FC-72 and R-123 nucleate boiling performance using a 15.6 mm diameter cylindrical surface with prism-shaped, nearly 0.9 mm tall microfins with a pitch of nearly 1.4 mm. The heating surface was finished with a microporous coating of aluminum brushable ceramic. Nucleate HTC values between 50% and 60% higher than those achieved using an uncoated boiling surface at imposed heat fluxes below 5 W/cm^2^ were reported. Nevertheless, this trend reversed at heat fluxes above 10 W/cm^2^, where the HTC and CHF values were lower than those obtained with an uncoated surface. This last trend was explained by the researchers as being the result of an appreciable enhancement in the bubble generation on the coated heating surface due to the vapor trapping in the spacing between fins. Furthermore, the authors Rioux et al. [120] and Li et al. [121] developed a hierarchical multiscaled boiling surface with copper macro-studs of sintered particles forming microporous structures. The combined enhancement approach yielded higher enhancements in the nucleate water HTC and CHF when compared to those obtained only using the individual enhancement procedures separately. It can be stated that the combination of the various enhancement techniques did not imply the achievement of one overall boiling heat transfer enhancement equal to the sum of the enhancements derived from the individual techniques. Figure 14 schematically presents the liquid–vapor interaction on a heating surface that suffered laser processing, where its microscale fins extended the available heat transfer surface area, and the pores act as preferential active nucleation sites and vapor traps stimulating the heat transfer enhancement. Additionally, the combined usage of microfins and pores offers extra liquid pathways to the nucleation sites and enhances the liquid–vapor interaction over the heating surface, leading to a steady fluid supply to the nucleation sites and improved heat transfer performance.

##### Fins and Particles or Multiscale Structures

The authors Chu et al. [122] evaluated the nucleate boiling behavior with two different hybrid enhancement surfaces on a silicon substrate. One experimented heating surface possessed 5 to 10 µm diameter and 10 µm tall silicon microfins with 14 nm sized silica particles deposited on them through electrophoresis. The other heating surface had 30 to 35 µm of diameter and 35 to 68 µm tall electroplated copper microfins covered with zinc oxide nanostructures having a non-dimensional roughness ranging from 3.6 to 13.3. The highest CHF enhancement of 200% was obtained using the second surface, with the highest non-dimensional roughness value of 13.3. Furthermore, the researchers Rahman et al. [123] synthesized different microfins on silicon substrates by deep reaction ion etching, which were coated with nickel nanostructures by a bio-templated technique using the self-assembly and metallization of tobacco mosaic virus structure. By measuring the fluid flux wicked into the surface structures, the investigation team demonstrated that capillary wicking was the main factor impacting the water CHF of structured superhydrophilic surfaces. The authors also proposed Equation (7) for the CHF as a function of the non-dimensional wicking number W_i_:

(4) $$\frac{q_{CHF}^{\prime \prime }}{q_{CHF,Zuber}^{\prime \prime }}=1+W_{i}$$

where $q_{CHF,\;Zuber}^{\prime \prime }$ is the predicted CHF value from the Zuber model [124], which can be expressed by Equation (8):

(5) $$q_{CHF,Zuber}^{\prime \prime }=0.131\rho_{g}h_{fg}{\left[\sigma_{g}\left(\rho_{f}-\rho_{g}\right)/\rho_{g}^{2}\right]}^{1/4}$$

W_i_ is the ratio of fluid flux wicked into the surface structures to critical vapor flux escaping from the surface and can be expressed through Equation (9), where V″ is the measured wicked flux:

(6) $$W_{i}=\frac{V^{\prime \prime }\rho_{f}}{\rho_{g}{\left[\sigma_{g}\left(\rho_{f}-\rho_{g}\right)/\rho_{g}^{2}\right]}^{1/4}}$$

A follow-up work conducted by Rahman and McCarthy [125] evaluated the pool boiling performance for copper surfaces presenting multiscaled enhanced structures, including channel arrays with lengths between 300 and 3000 µm, coatings with thickness ranging from 50 to 50 µm, and the combined employment of microchannels and nanocoatings. The microchannels were produced by electric discharge machining, and the nanostructures were produced by hydrothermal oxidation. Because of the enhanced surface wicking effect, the nanocoatings increased the water CHF value but decreased its nucleate HTC, which was caused by the suppression of the nucleation process of the bubbles. In addition, the microchannels enhanced the nucleate HTC value and the CHF. The hybrid surface produced by both enhancement routes showed the best performance, achieving high values of HTC and CHF of 461 kW/m^2^·K and 313 W/cm^2^, respectively.

##### Hydrophilic, Hydrophobic, and Biphilic Surfaces

**Figure 15:**
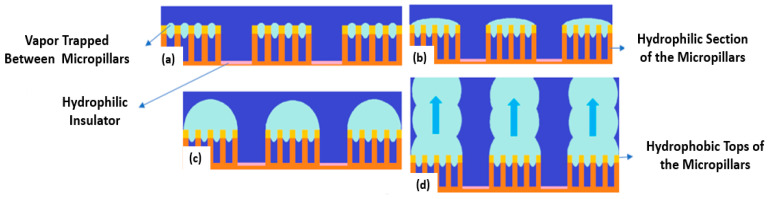
Schematic diagram of the nucleate boiling process stages using a biphilic heating surface with micropillar arrays: (**a**) vapor bubbles generated between the micropillars; (**b**) vapor bubbles merge, forming larger bubbles; (**c**) isolated bubbles at low superheats; (**d**) jets of columns at high superheats. Adapted from [133].

The already published experimental studies reveal that hydrophilic and hydrophobic surfaces possess a considerable propensity for nucleate pool boiling heat transfer enhancement. The CHF decreases in cases where hydrophobic boiling surfaces are employed, whereas hydrophilic boiling surfaces normally exhibit higher nucleate HTC and CHF values. The verified progress regarding the alteration of the surface energy of the heating surface creates a new chance for pool boiling heat transfer performance enhancement through the modification of the contact angle after the inclusion of nanostructures on the heating surface, which form a layer with great porosity during the boiling process. This last layer can alter the heating surface wettability and improve the CHF and the overall nucleate boiling behavior. All the involved mechanisms strongly suggest that surface wettability modification is a practical methodology very suitable for effectively enhancing the heat transfer capability under pool boiling scenarios. Moreover, the researchers Betz et al. [126] noted that a hydrophilic heating surface can delay the vapor film formation and the vapor film boiling regime, whereas a hydrophobic boiling surface can augment the existing number of active nucleation sites. The authors analyzed different heating surface patterns, including hydrophilic and hydrophobic, for pool boiling heat transfer enhancement purposes and reported improved values of the nucleate HTC and CHF for all the experimental surface patterns. Maximum enhancements of 65% for HTC and 100% for CHF were reported compared to the HTC and CHF obtained with a bare boiling surface. Moreover, the authors Phan et al. [127] used metal organic chemical vapor deposition (MOCVD), PECVD, and nanoparticle deposition during the nanofluid boiling process to produce surfaces with different wettability to evaluate the effect of the contact angle on the water nucleate boiling heat transfer. The obtained hydrophobic surfaces lowered the incipience superheat but hindered the detachment of bubbles and caused the spreading and coalescence of the vapor bubbles at high heat fluxes, resulting in the formation of a vapor blanket over the heating surface. The produced hydrophilic surfaces provoked an increase in the bubble departure radius, but the bubble departure frequency decreased with increasing wettability. Also, a surface with contact angles ranging from 45° to 90° exhibited a decrease in the HTC with a decreasing contact angle. Nevertheless, surfaces with contact angles lower than 45° presented improved nucleate boiling heat transfer behavior with a decreasing contact angle. The authors revealed that the highest HTC value was achieved using a surface with a near-zero contact angle. In addition, the researchers Kim et al. [128] showed that the wettability change of the titania coating with increasing temperature had an impact on the nucleate HTC and CHF values. The titania-coated surface had a hydrophobic character at low temperatures that changed to hydrophilic with increasing temperature. For example, increasing the surface temperature from 100 to 200 °C provoked a decrease in the water contact angle from 83.1 to 32.7 °C. Also, the authors Jo et al. [129] used hydrophilic silica coatings and hydrophobic Teflon coatings on boiling surfaces made of silicon to evaluate the influence of surface wettability on the pool boiling heat transfer behavior. The hydrophobic heating surfaces revealed to be capable of offering ameliorated water nucleate boiling heat transfer performance at low heat fluxes, and the hydrophilic boiling surfaces exhibited better performance at high heat fluxes. A heterogeneous surface composed of hydrophobic dots on a hydrophilic substrate offered better boiling heat transfer performance than either the boiling homogeneous hydrophobic or the boiling homogeneous hydrophilic surfaces. Nevertheless, the CHF for the heterogeneous surface was identical to those provided by the homogeneous hydrophilic surface. Moreover, the authors Betz et al. [130] examined hydrophilic, superhydrophilic, hydrophobic, superhydrophobic, biphilic, and superbiphilic coated surfaces. The superhydrophilic surfaces had a near-zero water contact angle, and the superhydrophobic ones exhibited a water contact angle higher than 150°. The biphilic surfaces were composed of hydrophilic and hydrophobic regions, and the superbiphilic surfaces were produced by juxtaposing superhydrophilic and superhydrophobic regions. It should be emphasized that the improved nucleate HTC and CHF values were achieved using biphilic surfaces compared to those obtained using surfaces of homogeneous wettability. The high values of CHF over 100 W/cm^2^ and HTC over 100 kW/m^2^·K were reached employing the superbiphilic heating surfaces. Additionally, the authors Choi et al. [131] proposed a practical methodology for producing a hybrid wettability surface by printing hydrophobic polymer dot arrays with a contact angle of 110° on a stainless steel substrate, which was then followed by the inclusion of hydrophilic zinc oxide nanostructures with 20° of contact angle and formed by micro-reactor-assisted nanomaterial deposition. This enhanced surface achieved a three-fold improvement in the water nucleate boiling HTC value. Also, the authors Bertossi et al. [132] developed a wettability switchable heating surface employing thermo-responsive polymers coated on a stainless steel surface. When the temperature of the heating surface reached the polymer switching temperature, which is slightly higher than the saturation temperature of the working fluid, the polymer wettability transitioned from hydrophilic to hydrophobic, therefore promoting vapor bubble nucleation. During the bubble growth, the heat conducted near the triple-phase contact line induced a localized decrease in the polymer temperature, which switched the wettability back from a hydrophobic to a hydrophilic nature. Nevertheless, the water-nucleate HTC enhancement was limited to a moderate, nearly 20% increase. Additionally, in the study conducted by the authors of [133], the nucleate boiling heat transfer enhancement was evaluated using superhydrophilic, hydrophobic, and micro-patterned hydrophobic/superhydrophilic boiling surfaces. For this purpose, the authors air-sprayed stainless steel foils with a coating composed of silica and PDMS with high hydrophobicity due to their hierarchical structure and the incorporation of a hydrophobic polymer. The coating was machined with a pulsed Nd:YAG laser to produce biphilic patterns. The homogeneous superhydrophilic surface possessed the greatest CHF enhancement, superior by 350% compared to that provided by the uncoated stainless steel heating foil. The researchers reported that the increased wettability reduced the bubble contact diameter, allowed a higher density of active preferential nucleation sites, and delayed the dry-out action. An opposite tendency was observed when using the hydrophobic heat transfer surface, where the vapor film covered the surface after the boiling incipience. Nevertheless, this surface stimulated a high nucleate HTC value at reduced imposed heat fluxes as the first bubble appeared on the surface at a superheat degree of less than 1K. The biphilic surfaces with square hydrophobic spots of 4, 1, and 0.0625 mm^2^ area exhibited a CHF value up to 200% higher than that provided by the uncoated stainless steel boiling foil. The best nucleate HTC value of 51.2 kW/m^2^ was achieved using the boiling surface with the smallest hydrophobic spots, given that those reduced the diameter of the vapor bubbles and increased their nucleation frequency. The surfaces with larger hydrophobic spots fostered boiling incipience and exhibited higher HTC values at low imposed heat fluxes. With increasing heat flux, the vapor film started to cover the hydrophobic regions, reducing the heat transfer capability of the system. Figure 15 presents the fundamental steps of bubble dynamics on a biphilic surface with micropillar arrays.

**Figure 16:**
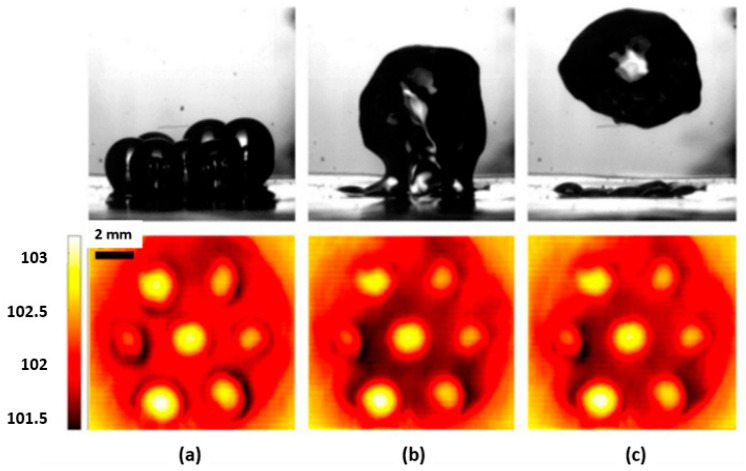
Synchronized images and thermographical images of the bubbles generated in seven hydrophobic spots with a spacing equal to the diameter of the bubbles. The (**a**–**c**) conditions refer to before, during, and after coalescence, respectively [75].

Furthermore, in the work conducted by the researchers Freitas et al. [75], the dynamic evolution of the pool boiling bubbles was studied using biphilic heat transfer surfaces and an aqueous silver nanofluid at 1 wt.%. Figure 16 presents some synchronized images of the mentioned bubble dynamics analysis for an imposed heat flux of 1290/m^2^.

**Figure 17:**
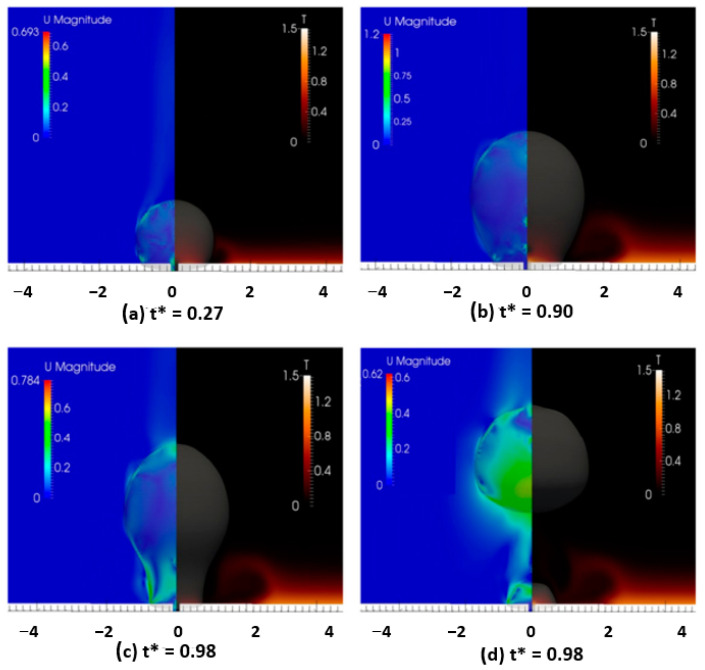
Velocity magnitude and temperature during bubble growth for superhydrophobic regions with 1.5 mm of diameter: (**a**) bubble growth at t* = 0.27, (**b**) bubble growth at t* = 0.90, (**c**) bubble necking at t* = 0.98, (**d**) bubble growth at t* = 1.00 [75].

Also, the velocity magnitude and temperature profiles during the bubble growth stage were investigated for an imposed heat flux of 1290 W/m^2^ and using water as the operating fluid. Figure 17 presents some of the results of the velocity, magnitude, and temperature during bubble growth.

Generally, pool-boiling-related studies using biphilic surfaces have proved that hydrophilic surfaces with an array of hydrophobic dots are very effective in improving the nucleate boiling heat transfer capability. The biphilic and superbiphilic boiling surfaces have confirmed promising results, given that the size and number of hydrophobic spots determine the active nucleation site density and the diameter of the vapor bubbles. The surface hydrophilic regions ensure the suction of the working thermal fluid toward the active nucleation sites and prevent the coalescence of the bubbles. Nonetheless, the main conclusion that can be easily retrieved from a comprehensive survey is the scarcity of sufficiently large, published databases of concepts regarding the use of pool boiling heating biphilic surfaces. Therefore, the durability of the wettability-modified surfaces in harsh pool boiling regimes is still a limitation to be overcome, and further experimental studies are highly recommended to offer improved knowledge of the potential effectiveness, reliability, durability, and overall cost of the biphilic boiling surfaces and of their enhanced nucleate pool boiling heat transfer performance.

##### Surface Roughening

The surface roughness has a profound impact on the nucleate boiling heat transfer behavior, including single-phase and two-phase convection and radiation [134]. Thus, enhancing the roughness of the heating surface by sandblasting, chemical etching, mechanical roughening, and induction of artificial surface cavities are commonly followed approaches for ameliorating nucleate pool boiling performance. The reason behind such enhancements is the higher number of active nucleation sites on the heating surface. Furthermore, the researchers Kim et al. [135] found a profound dependence of the water CHF on the boiling surface roughness. The CHF was found to increase from 77.5 W/cm^2^ for a smooth surface with an average roughness value of around 0.04 µm to 162.5 W/cm^2^ for a rough surface with an average roughness value of nearly 2.4 µm. Such enhancement was ascribed by the research team to the improved capillary wicking effect of the rougher heating surface. In addition, the authors proposed Equation (4), which accounts for the wicking effects:

(7) $$q_{CHF}^{\prime \prime }=0.811\left(\frac{1+cos\alpha }{16}\right)\cdot {\left[\frac{2}{\pi }+\frac{\pi }{4}\left(1+cos\alpha \right)+\frac{351.2cos\alpha }{1+cos\alpha }\left(\frac{R_{a}}{S_{m}}\right)\right]}^{1/2}\cdot \rho_{g}h_{fg}{\left[\frac{\sigma g\left(\rho_{f}-\rho_{g}\right)}{\rho_{g}^{2}}\right]}^{1/4}$$

where α is the static contact angle, and S_m_ is the mean spacing between the heating surface roughness peaks. Moreover, despite many published works demonstrating the benefits of the surface roughening enhancement route, this scheme is still not widely adopted on a large scale. The main reason behind the absence of commercial implementation of roughened enhanced surfaces is the very fast aging effect of the surface features, normally lasting only for a few hours, during which the pool boiling heat transfer behavior will gradually deteriorate until it is like that obtained using a plain, bare heating surface. One demonstration of this effect was the study performed by the authors Chaudhri and McDougall [136], in which they evaluated the long-term boiling heat transfer performance using boiling surfaces having parallel scratches with 0.025 mm of width. The researchers also showed that the heat transfer capability enhancement employing perchloroethylene and isopropyl acetate was only temporary, and the heat transfer performance after boiling for hundreds of hours abated back to those induced by a plain, bare heating surface. It is therefore imperative that further studies on the surface roughening enhancement approach entail an accurate assessment of the surface aging effects. Apart from that, the surface roughness is normally characterized by the average roughness value R_a_ or by the effective heat transfer area ratio or heat transfer area-to-projected ratio r in the previous published studies. One such example is the study conducted by the researchers Moita et al. [108], who found that surfaces with a larger r could not assure a greater nucleate HTC. The authors demonstrated that a larger r could in fact increase the liquid–solid contact area, hence improving the nucleate HTC, but at the same time, it may cause the early formation of a vapor blanket due to the coalescence of the bubbles. This would degrade the nucleate HTC and, even overcome the benefits of the increased liquid–solid contact area. Also, some researchers found an increased number of active nucleation sites on heating surfaces with larger average roughness, which is normally held responsible for the nucleate HTC enhancement. The main difficulty in fully understanding the impact of the heat transfer surface roughness on the pool boiling process arises from the inconsistent related experimental data, which happens from the different roughening techniques, measurement uncertainties, and different factors like the type and thermophysical characteristics of the working fluid, surface material, and wettability. In this direction, the authors Kim et al. examined the effect of the heating surface roughness on the pool boiling heat transfer behavior using a superhydrophilic [137], moderately wetted [135], and hydrophobic surface [138]. In cases where superhydrophilic and moderately wetted surfaces were employed, the nucleate HTC value increased with increasing boiling surface roughness. Nonetheless, using the hydrophobic boiling surface demonstrated that the HTC of a rougher surface was initially higher than the corresponding HTC of a smoother one but decreased rapidly at moderate and high heat fluxes, leading to an early occurrence of the CHF. Such a trend was explained by the researchers as being the result of the early formation of a vapor blanket on the hydrophobic surface, as it was already confirmed by the researchers Moita et al. [108]. Other authors have pointed out the need for using the same consistent surface roughening technique when studying the effect of the boiling surface average roughness on the pool boiling process [139] given that heat transfer surfaces prepared by certain roughening techniques have a higher tendency to bubble nucleation than others prepared by different techniques, regardless of the average surface roughness final values. In addition, the surface roughness parameters R_a_ and R_q_ are statistically obtained values that are unable to accurately translate the real texture of the surface, especially concerning the number of active nucleation cavities. Hence, a smooth heating surface with a small R_a_ or R_q_ can have a great number of cavities suitable for nucleation. Nonetheless, such approaches follow the simplistic treatment of the surface texture and wettability. Thus, the quantitative prediction of the nucleation site total number is still a much more difficult or even unrealizable process; hence, authors must still characterize the surface roughness based on statistical roughness parameters. Furthermore, some authors have studied the impact on the nucleate boiling heat transfer capability of the tridimensional roughness. That was the case of the study conducted by the authors Fan et al. [140], who aimed to investigate such an impact on water pool boiling using copper surfaces. In this direction, different enhanced surfaces made of copper were fabricated by polishing and femtosecond laser machining techniques, presenting an average roughness between 0.045 and 1.35 μm. In view of the obtained experimental data, the researchers proposed a correlation accounting for the tridimensional roughness parameter S_a_. A bidimensional roughness profile can be separated into large-scale waviness and short-scale roughness. In addition,, the average height of the profile, waviness, and roughness can be denoted as P_a_, W_a_, and R_a_, respectively. Nonetheless, in the tridimensional case, instead of using the bidimensional parameters, the roughness is defined by the parameters S_ap_ and S_a_, which are equivalent to the referred P_a_ and R_a_. It was observed that a rougher surface had a higher nucleate HTC than a smoother one only if both surfaces suffered the same roughening procedure and if the surface roughness was characterized by the S_ap_ parameter. Hence, a laser-machined surface with S_ap_ equal to 3.4 μm could deteriorate the HTC when compared with a surface polished by 180-grit sandpaper with S_ap_ around 1.3 μm. In view of the obtained experimental results, the authors deduced the following conclusions: (i) The contact angle of the sandpaper polished surfaces increased initially with increasing surface roughness and then remained constant as the surface roughness further increased. This trend was interpreted by the authors as being the result of the spreading of the sessile droplet on the contact angle measurements. A rougher surface with larger and deeper scratches could block the spreading of the droplets, and, therefore, the authors noted that the contact angle increased with increasing surface roughness. Nevertheless, this increase did not occur for a S_a_ superior to around 0.6 μm, beyond which the obstruction of the droplet spreading reached a threshold. For the laser-machined surfaces, the contact angle was around 153°, and the surface roughness had only a minor effect on it. (ii) The boiling curves shifted to the left as the surface roughness parameter S_a_ became larger, regardless of the surface preparation procedure. The surface roughening procedure had only a negligible impact on the nucleated HTC. The CHF was affected by the surface roughness and surface roughening technique. (iii) The rough surfaces enhanced the nucleate boiling heat transfer behavior by providing more nucleation sites, and at the bubble departure stage, decreasing their diameter and increasing their departure frequency. The number of nucleated bubbles increased with increasing imposed heat flux. (iv) The proposed numerical correlation accurately predicted the HTC with different S_a_.

##### Extended Surface

The development of finned heating surfaces with rectangular or square fins is a commonly employed procedure to achieve nucleate boiling heat transfer enhancement. The primary beneficial characteristic of this methodology is the extended heat transfer area, and the main challenge related to it is the optimization of the fin size and spacing between the fins to reach an optimal thermal performance. In this sense, the authors Rainey and You [118] observed that the FC-72 nucleate boiling heat transfer behavior using boiling surfaces having 1 mm sized fins and 1 mm of spacing between them was profoundly improved by the increase in the fin height by up to 5 mm. Above this height, the temperature of the fin tip becomes too low to maintain the boiling process running, and reducing the available area for boiling heat transfer, hence, providing no extra heat transfer capability increase. On the other hand, the researchers Chu et al. [141] found that the water CHF using finned enhanced heat transfer surfaces increased in cases where the non-dimensional roughness increased from nearly 1.8 to around 5.9. The authors modified the model introduced by Kandlikar [142] to better account for the surface tension from the bubbles and proposed Equation 5 for the CHF using finned improved surfaces:

(8) $$q_{CHF}^{\prime \prime }=\frac{1+cos\alpha }{16}{\left[\frac{2}{\pi }\left(\frac{1+\alpha_{m}}{1+cos\alpha }\right)+\frac{\pi }{4}\left(1+cos\alpha \right)cos\theta \right]}^{1/2}\cdot \rho_{g}h_{fg}{\left[\sigma g\left(\rho_{f}-\rho_{g}\right)/\rho_{g}^{2}\right]}^{1/4}$$

where r^+^ is the non-dimensional roughness, α is the fluid contact angle on a smooth surface, α_rec_ is the receding contact angle of the fluid on a smooth surface, and α_m_ is the fluid modified contact angle on a finned surface, which can be expressed by Equation (6):

(9) $$\alpha_{m}=r^{+}cos\alpha_{rec}$$

Accordingly, Kandlikar [143] developed a finned modified surface in which the base of the fins sharp corners promoted the nucleation locally, and as the bubbles moved along the heating surface induced the fluid to flow over the fins toward the preferential nucleation corners. The authors noted an 8-fold enhancement in the HTC and a 2.5-fold enhancement in the CHF for water compared to those obtained with a smooth copper heating surface. Moreover, the authors McNeil et al. [144] conducted boiling experiments using the R-113 coolant and water as the thermal working fluids and a copper heat sink with in-line arranged pin fins with a square cross section of 1 mm^2^ of area, a height of 1 mm, and a spacing between them of 2 mm. The goal of the study was to determine the influence of the substrate thickness and the effectiveness of the pin fins on the nucleate boiling heat transfer behavior. Also, it should be stated that the effective heat transfer area ratio becomes the geometric heat transfer area ratio when the fin efficiency is equal to 1, and, in this case, the geometric ratio was 1.75. The path through the pin fins became less effective as the thermal conductivity of the heating surface decreased, and consequently, a larger heating surface thermal conductivity was required for the extended surface to be useful, but a uniform heat flux cannot be achieved with a high thermally conductive heating surface. The spike in the heat flux at the heat sink inlet was diminished by decreasing the surface thermal conductivity. Nonetheless, the extended surface was rendered ineffective. Also, the effect of the substrate thickness was assessed too, altering the total surface thickness while sustaining one half thickness made of copper and the other half thickness made of aluminum. As the surface thickness decreased, the effective area ratio remained constant, and the heat flux became more uniform. Nonetheless, even at a thickness of 1 mm, it achieved only a non-uniform heat flux with distortions near the inlet and outlet of the heat sink. Finally, it can be stated that further studies are highly recommended to give some guidelines about the optimal fin size, geometry, pitch, and configuration for different types of thermal fluids, operating conditions, and surface thermal conductivity, thickness, and orientation.

##### Bi-Conductive Surfaces

**Figure 18:**
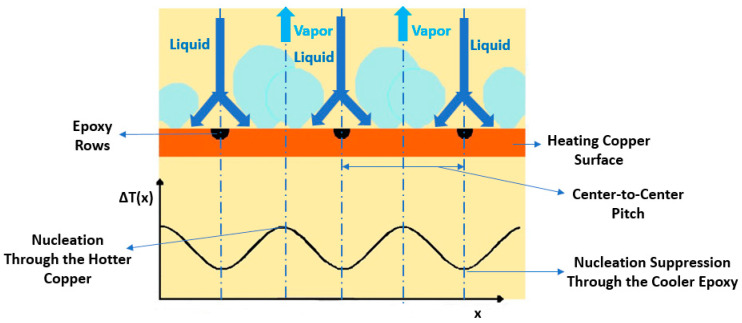
Schematic diagram and plot regarding the nucleation and liquid/vapor separation on a bi-conductive surface. Adapted from [147].

The use of bi-conductive surfaces provides a promising prospect for the enhancement of nucleate boiling heat transfer. Though the advantages of bi-conductive surfaces in the pool boiling heat transfer capability enhancement were already confirmed, the enhancement mechanism has not yet been completely well understood because of the lack of experimental data related to temperature, velocity, and two-phase interface evolution. Additionally, the researchers Deng et al. [145] analyzed the pool boiling phase change heat transfer on bi-conductive surfaces that were composed of a high thermally conductive substrate with embedded low thermally conductive inserts. The authors replicated the stages of the bubble dynamics regarding the nucleation, growth, and departure in the bi-conductive surface and assessed the evolution of the temperature and fluid flow during the pool boiling process. In view of the experimental results, the authors deduced the following concluding remarks: (i) The nucleate pool boiling enhancement mechanism on the bi-conductive heating surface relied on the induced pinning of the vapor bubble contact line. In other words, restricting the contacts of the bubble prevented the coalescence of the bubbles and ensured that the bubbles could only grow vertically. Since the contact area of the restricted single bubbles and the surface were limited, the detachment frequency of the vapor bubbles increased considerably, and therefore, the HTC and CHF values of the bi-conductive surfaces was also enhanced. (ii) The boiling process was optimized when the embedding spacing of the insets was like the capillary length of the working fluid. A spacing that was too small resulted in bubble coalescence before growth, and a spacing that was too large led to multiple bubble generation and coalescence. Both of these behaviors could result in the formation of a localized vapor film, which in turn may reduce the boiling performance. (iii) Increasing the width of the insets improved the HTC at low superheat degrees, but it also led to the CHF enhancement, while changing the depth of the insets had only a minor influence on the nucleate boiling heat transfer capability of the bi-conductive heating surfaces. (iv) The bi-conductive surfaces stimulated the CHF by embedding a poor thermally conductive material into the highly thermally conductive bulk substrate and, hence, exhibited a reliable nucleate boiling heat transfer capability enhancement. In the experimental work carried out by the authors Heidary et al. [146] the effect of pitch, width, and depth of low-conductive channels was investigated on water pool boiling using a bi-conductive heat transfer surface. In this direction, copper surfaces were grooved by wire electrical discharge. The grooves were filled with a mixture of epoxy and hydrophilic silica aerogel to accelerate water replenishment to the heating surface. The experimental results indicated that the surface with 2.5 mm channel pitch, 0.5 mm width, and 0.3 mm depth reached a heat flux of 103.9 W/cm^2^ and a nucleate HTC of 7.6 W/cm^2^·K, which represents 62% and 58% enhancements, respectively, compared to those provided by a plain boiling surface. The ideal selection of the channel pitch resulted in a 21% decrease in the bubble departure diameter. Also, increasing the channel width to an optimal value of 1.5 mm led to an enhanced HTC peak value of 146% compared to that achieved by using a plain surface at low heat fluxes. Additionally, increasing the depth of the channels could be inversely correlated with the improvement in pool boiling performance, given that the thermal conductivity of the heating surface decreased. Moreover, the researchers Rahman et al. [147] reported a boiling enhancing heat transfer procedure by incorporating low-conductivity materials at the heating surface/working fluid interface. The bi-conductive surfaces were composed of some rows of low conductive epoxy embedded into high-conductive copper substrates. The bi-conductive surfaces were fabricated with a varying number of epoxy divisions per centimeter, N. In total, six different bi-conductive surfaces were fabricated with N = 2, 4, 6, 8, 10, and 12 cm^−1^. The embedded epoxy divisions had a width of around 420 μm, a depth of nearly 290 μm, and center-to-center pitches between nearly 1 mm and 3.7 mm. The authors created spatial variations in the surface temperature during the boiling process, and by adjusting these variations to coincide with the capillary length, the authors found 5-fold and 2-fold increases in the nucleate HTC and CHF values, respectively. The enhancements were attributed to the ability of in-plane variations in the wall superheat to separate the liquid and vapor flows, leading to a high HTC and a delay in the CHF. Figure 18 schematically illustrates the nucleation and liquid–vapor pathway segregation on a boiling bi-conductive surface.

By reducing the superheat temperature in the vicinity of the low-conductivity materials, the epoxy divisions avert nucleation and remain wet during the nucleate boiling process. While these effects diminish the heat transfer rate near the epoxy divisions, they increase the overall heat transfer rate over the entire heating surface by imparting spatially ordered and enhanced bubble dynamics. The epoxy divisions also promoted the coalescence-driven departure of laterally merging bubbles. As the two bubbles merged, the underlying epoxy division between them remained wet. A thin liquid layer remains on the epoxy surface, and the non-wetted base areas below each bubble do not merge, instead collapsing or moving during the boiling process. The resulting bubble deforms due to the surface tension and is quickly removed from the heating surface. The epoxy inhibits the inward lateral merging of the non-wetted bases and facilitates the replenishment of the thermal fluid existent underneath the bubbles, thus promoting the bubble departure frequency. At an imposed heat flux of 91 W/cm^−2^, the required wall superheat more than doubled across the experimental heating surfaces, varying from 9 to 19 K. At the CHF, each boiling surface was operating at heat fluxes between 91 and 230 W/cm^−2^ and at wall superheats between 11 and 19 K and, for all cases, the maximum nucleate HTC value was confirmed using bi-conductive surfaces with the pitch equal to the capillary length. The 5-fold heat transfer rate enhancement at a given superheat was interpreted by the researchers as being the result of the bubble nucleation, growth, and departure processes. Also, the non-conductive epoxy ordered the vapor and liquid flows, which aid in supplying cool liquid to the surface and promote the departure of the bubbles. For the N = 2 cm^−1^ and N = 4 cm^−1^ experimental designs, the heat transfer rates steadily increased compared to those obtained with a bare heating surface. However, for N ≥ 6 cm^−1^ experimental designs, the vapor bubbles grew to diameters wider than the pitch between the epoxy divisions before they departed from the surface. Such an effect prevented the counterflow of cooler liquid returning to the surface and disrupted the ordering of the fluid flow pathways. Moreover, it was verified that there was a decrease in the nucleate HTC and CHF values of approximately 25% for the N ≥ 6 cm^−1^ experimental configurations compared to those achieved using a bare copper boiling surface. By using the temperature gradients to order the location of the active nucleation sites and promoting the formation of distinct paths for the liquid and vapor flows, the HTC increased from 41 to 210 kW/m^2^·K at a superheat of 11 K and the CHF increased from 116 to 230 W/cm^2^. With this active boiling enhancement technique, the enhancements are mainly derived from the thermal conductivity bulk property, as opposed to surface properties or surface structures. Unlike surface coatings, this mechanism is not susceptible to material degradation and effectiveness concerns over time, nor is it reliant on thermal fluid types and characteristics to promote boiling, as it is required for surfaces with low surface energy materials.

#### 2.2.3. Fluid Flow Enhancement

##### Displaced Enhancement

**Figure 19:**
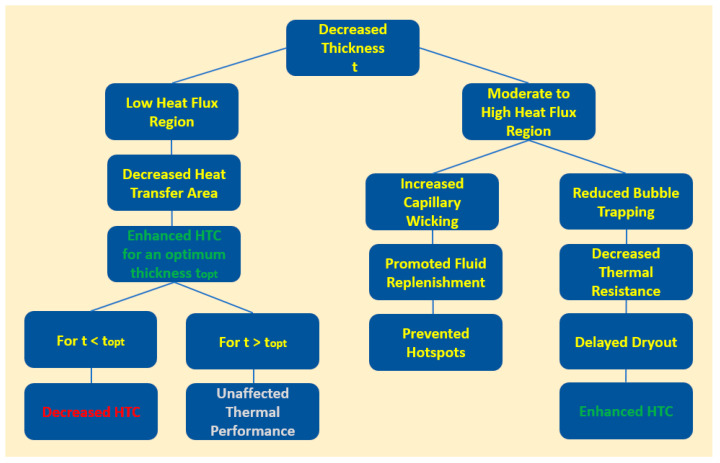
Diagram summarizing the impact of a metal foam attachment thickness on the pool boiling of a commercial coolant. Adapted from [149].

The heat transfer enhancement technique known as displaced enhancement involves the use of foams and metallic mesh attachments, static mixers, screen lamination, and the inclusion of different kinds of objects fitted over the heating surface, like plates, rings, disks, manifolds, and objects and structures of complex geometries. Improved fluid mixing, effective liquid–vapor pathway separation, and pressure recovery are some of the merits of this nucleate boiling heat transfer enhancement technique. The use of a metallic foam with a porous fraction greater than 90% attracted the attention of the researchers because of its lightweight and its great heat transfer enhancement capability. For instance, the authors Xu et al. [148] evaluated the acetone pool boiling beneficial features of using a copper foam. The main factor influencing heat transfer behavior was found to be the total number of pores per inch (PPI). Low PPI foams showed improved heat transfer at small liquid superheats, whereas high PPI foams were more performant with moderate to large superheat degrees. Additionally, the thermal performance and the nucleation site density increased with a higher foam layer thickness, but with a thicker foam, the resistance to the vapor venting also increased. The diagram in Figure 19 summarizes the effects derived from the thickness of a metal foam on the HFE-7100 refrigerant pool boiling.

**Figure 20:**
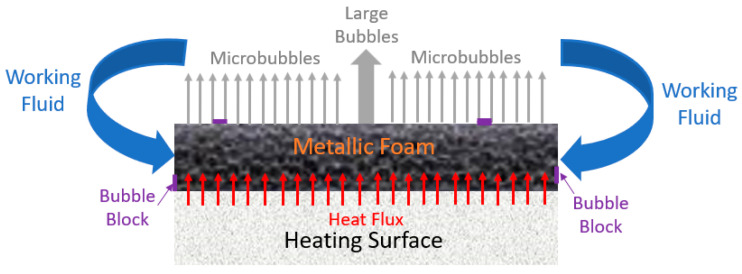
Schematic illustration of the nucleate boiling mechanism using a metallic foam attachment. Adapted from [152].

In addition, the researchers Yang et al. [150] studied a copper foam welded to a copper heating substrate under pool boiling conditions. The authors reported that the heat transfer surface reduced the incipience superheat, enhanced the CHF value up to three times, and appreciably reduced the onset of the nucleate boiling using water as operating fluid when compared to those provided by a plain copper boiling surface. The authors obtained a peak in the heat transfer enhancement employing a PPI of 60 and deduced that the boiling performance depended on both the PPI and foam layer thickness. Apart from that, the authors Xu and Zhao [151] proposed the application of a non-uniform metal foam with high pore density near the heating surface and low pore density away from it, thereby enhancing the water nucleate boiling behavior. Figure 20 illustrates the nucleate boiling enhancement mechanism of a metallic foam attachment.

In the experimental work performed by the authors Hayes et al. [153], diverse hollow conical structures were printed and placed above the heat transfer aluminum surface to provide a better liquid–vapor separation. The authors hypothesized that buoyancy and fluid inertia forces could be very useful in regions over the boiling surface to implement an ordered and separated flow of the liquid and vapor phases. The flow of liquid and vapor above the heat transfer surface was controlled by placing the hollow conical structures produced by metal additive manufacturing over the heating surface. The hollow conical structures were perforated at the top and sides to allow the vapor bubbles generated under the structures to be quickly removed while, at the same time, enabling the bulk liquid to undertake the heating surface rewetting action. As the liquid boiled and vapor accumulated inside the structures, independent liquid and vapor flow pathways were generated at the holes of the conical structures. The efficient removal of the vapor and the short fluid flow length over the heating surface provided an enhancement in the nucleate boiling heat transfer behavior. Hence, the boiling performance enhancement from these structures was due to the flow field modulation and the secondary boiling outside the hollow conical structures caused by the increased population of active nucleation sites. The experimental results revealed that 74% of the boiling enhancement achieved with these structures was the result of the convective heat transfer action. The boiling performance was further increased using the same structures over microchannels to improve the heat transfer capability of the nearby entrance region. By the combined usage of several miniaturized HCS with microchannels, it was possible to achieve a nucleate HTC value of 190 kW/m^2^·K, corresponding to a 4-fold increase compared to the HTC provided by a plain aluminum heating surface. Also, this study confirmed the ability of additive manufacturing to create structures that use macro-convection to ameliorate the nucleate boiling heat transfer behavior. Additionally, the authors Chauhan and Kandlikar [154] developed a dual-tapered evaporator in a thermosiphon loop and achieved superior heat dissipation performance compared to that of water and air. An improved performance was achieved by the dual-tapered experimental configuration when compared to that provided by a single-tapered one due to the lower pressure drop across the boiling section as the fluid flow length is reduced by half. The concept of a dual-tapered microgap was extended to a water pool boiling system [155] where a CHF of 288 W/cm^2^ was achieved on a plain copper heating chip. A 2.3-fold enhancement in the HTC and CHF values was obtained by using the dual-tapered configuration. The vapor bubble squeezing and pressure recovery within the tapered microgap created a self-sustaining fluid pumping action, which promoted a stable two-phase flow and continuous surface rewetting at high imposed heat fluxes, thereby increasing the heat dissipation capability of the system. Moreover, a study was conducted by Chauhan and Kandlikar [156] to evaluate the pool boiling heat transfer behavior of a dual-tapered microgap using a dielectric liquid as the operating thermal fluid. The main objectives were to infer the impact of the taper angle and inlet gap height on the nucleate HTC and CHF values and to predict the HTC through a proposed model. In this direction, taper angles between 5° and 25° and inlet gap heights of 0.8 mm and 1.27 mm were employed together with the HFE-7000 commercial thermal fluid. A reduction in the CHF was observed with 5°, 10°, and 15° taper angles with both inlet gap heights due to the lower pressure recovery when using smaller taper angles. A higher nucleate HTC value was achieved with all taper angles compared to the HTC achieved with an experimental design without a manifold block. The HTC obtained for 20° and 25° taper angles with a 0.8 mm inlet gap height were 13.5 kW/(m^2^·°C), and 17.8 kW/(m^2^·°C) at a CHF of 30 W/cm^2^. For the 1.27 mm inlet gap height, the HTC achieved for 20° and 25° taper angles was 14.5 kW/(m^2^·°C). A 2-fold and 1.5-fold enhancement in the HTC was recorded using a 25° taper angle with a inlet gap height of 0.8 mm and an inlet gap height of 1.27 mm, respectively, compared to the experimental configuration without the inclusion of a manifold block. A model based on the homogeneous fluid flow model and boiling heat transfer correlation was used to predict the HTC for different configurations. For the cases where the pressure recovery balanced the pressure drops with sustained flow, the HTC values were predicted by the experimental data comparison. The maximum deviation between the predicted and experimental HTC values was 13.3% for the 15° taper with a 0.8 mm inlet gap height. The obtained experimental results indicated lower heat dissipation performance when employing smaller taper angles. The developed model indicated low pressure recovery for smaller taper angles; hence, the existence of unstable vapor bubble expansion was foreseen in the microgap, leading to a reduction in the CHF value. For higher taper angles, the pressure recovery balanced the pressure drop due to the friction factor, momentum change, and geometry. In such cases, there were achieved enhanced HTC values, and the model predicted well the heat transfer capability. The pressure recovery, along with the tapered microgap length and bubble squeezing, were the responsible mechanisms for the high heat dissipation performance using the tapered experimental configuration. Such factors provide stable fluid flow and liquid pumping within the microgap. The tapered gap microchannels provided an enhancement structure, modifying the flow field over the heating surface by allowing a unidirectional flow created by the bubble expansion forces.

##### Swirl Flow

**Figure 21:**
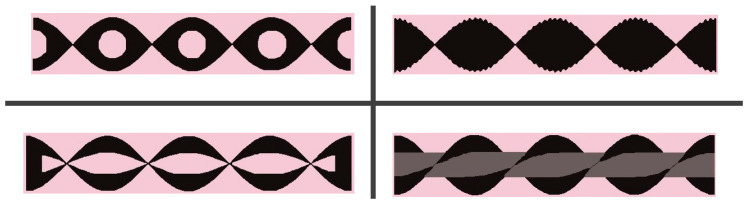
Typical sections of perforated and non-perforated swirl flow inserts. Adapted from [157].

The passive swirl flow-inducing technique using twisted tapes is one of the most effective methodologies for heat transfer enhancement purposes. Diverse experimental studies have been conducted with different twisted tapes to increase the rate of the nucleate pool boiling heat transfer. The twisted tapes are often metal strips that are twisted according to the intended shapes and dimensions and that are inserted in a heat transfer fluid flow field. Such tapes create turbulence in the fluid flow, imparting swirls on it, which may lead to an increase in the HTC value. The pitch-to-twist ratio is an important parameter, while studying the effect of the twisted tapes on the boiling heat transfer behavior improvement. The pitch of the tape is the distance between two points on a plane parallel to the axis of the tape. The twist ratio of the tape is the pitch-to-inner tube diameter ratio. The distance between the twisted tape and the boiling surface is also a relevant parameter for adopting the width of the twisted tapes to be used, given that an excessive distance may induce a by-pass flow, diminishing the heat transfer capability of the technique. The use of inserts like twisted tapes may cause fluid flow obstruction, induction of secondary flow, and flow portioning. The working fluid flow obstruction reduces the free flow area, pressure drop, and the effects of the viscosity. Along with this, the flow velocity also increases, and, in some cases, a secondary flow is generated, which in turn produces turbulence and promotes the effective mixing of the fluid in the flow region, which may increase the temperature gradient and the HTC value. Figure 21 presents a few examples of typical perforated and non-perforated swirl flow inserts used for nucleate pool boiling enhancement ends:

The researchers Nanan et al. [158] studied the impact of employing perforated helical twisted tapes on the nucleate boiling thermal performance and friction loss under an imposed heat flux. The perforated helical twisted tapes were obtained by punching helical twisted tapes with a prospect to reduce the friction loss of the fluid flow. The perforated tapes having diameter ratios (*d*/*w*) of 0.2, 0.4, and 0.6 were used, along with three different perforation pitch ratios (*s*/*w*) of 1, 1.5, and 2. The ratio of the helical pitch and twist was fixed at *p*/*d* of 2 and *y*/*w* of 3. The comparison experiments were carried out using a plain tube and tubes with helical twisted tapes. In view of the obtained experimental results, the authors deduced that the use of perforated helical twisted tapes decreased the friction loss compared to that imposed using non-perforated helical twisted tapes. The heat transfer, friction loss, and thermal performance factor increased as the diameter ratio decreased, and the pitch ratio increased. The ideal thermal performance factor of 1.28 was obtained with the perforated helical twisted tapes with *d*/*w* = 0.2 and *s*/*w* = 2.0. Additionally, the work conducted by the authors Ebrahimi-Dehshali et al. [159] evaluated the impact of the twisted tape fins (TTF) on the heat transfer capability using a flat surface under pool boiling conditions. Different plates with a few stainless-steel-made TTF ranging from 1 to 9 were developed and their water nucleate boiling performance was investigated. It was observed that the increase in the HTC of the plate with nine twisted tape fins was 15.5% compared to the HTC of a plain plate. The results indicated that by increasing the height of the twisted tape fins, heat transfer was enhanced. The nucleate HTC obtained with the highest TTF exhibited a nearly 23% enhancement compared to the HTC provided by the plain heating surface. The centrifugal force induced by the twisted tape fins resulted in the inclination of the bubbles and caused a faster bubble departure because of the faster liquid flow replacement. These effects led to a decrease in the coalescence of the bubbles and an increase in the number of nucleated vapor bubbles. The TTF exhibited a near surface enhancement mechanism when separated from the bubble–liquid pathways and a far surface enhancement mechanism by inducing a rising flow of vapor bubbles, and as the result, an improved nucleate boiling heat transfer behavior was achieved. Generally, the thermohydraulic behavior of the inserted twisted tapes with and without perforation was studied by various investigators, and it was found that the perforated twisted tapes were more reliable and effective when compared to the twisted ones without perforation and plane inserts. Also, the twisted tapes acted relatively better in laminar flow due to the efficient mixing of bulk flow. Diverse published studies compared the effects of the short- and full-length twisted tapes and confirmed that the short-length twisted tapes gave better thermal performance at a fixed pumping power. The use of twisted tapes creates large pressure drops provoked by the obstruction of the fluid flow when incorporating twisted tapes without perforation, but when employing twisted tapes with perforation, fewer pressure drops occurred due to the perforated holes themselves. Also, it was observed from the earlier available studies that the inclusion of twisted tapes in the heat exchanger systems was a successful methodology for the nucleate boiling heat transfer improvement. Accordingly, improvements in the Nusselt number, thermal performance, and friction factor were reported by including twisted tape inserts. Different types of geometries have been tested to check the heat transfer augmentation, and many useful correlations to determine heat transfer and friction have been proposed. The geometry and size of the perforation of the twisted tapes, coupled with the features of the device and fluid flow, are the main factors determining the thermohydraulic performance of the system. Also, certain new techniques, like the perforation of the inserted twisted tapes or the employment of broken twisted tapes, have also been proposed by the research community to develop and implement twisted tapes with and without perforation boiling heat transfer enhancement techniques. Nonetheless, further work is required to develop this technology and to obtain more useful insights and numeric correlations on the matter.

##### Surface Orientation and Gap Width

The implementation of a thermal management system usually dictates placing the heat dissipating device in vertical or horizontal orientations. In addition, a major part of the recent applications favored the development and implementation of small lightweighted systems. Such demands cause significant constraints on the allowable dimensions of the boiling chamber and fluid flow space around the heat dissipating equipment. Therefore, it relevant to infer the impact of the heating surface orientation and boiling confinement conditions on the nucleate pool boiling performance. Additionally, diverse authors like Geisler and Bar-Cohen [160] reported the advantageous features of the nucleate pool boiling enhancement of a vertical heating surface in a narrow fluid gap. Often, these beneficial features can be attributed to the sweeping action of the buoyancy forces, which promote bubble departure from the vertical heating surface. Moreover, the authors Mukherjee and Mudawar [161] studied the nucleate boiling process using a vertical heating surface mounted into a small boiling section along one of two parallel channel walls, allowing for gap width adjustments. The authors were able to separate the pathway of the vapor from the heating surface from the pathway of the replenished fluid with the aid of a pumpless cooling loop, which was composed of two vertical parallel tubes connected at the bottom and sharing a single reservoir atop. This configuration induced a clockwise motion of the thermal fluid, benefiting from the fluid density differences between the heavier fluid in the cold tube and the lighter two-phase mixture in the hot tube. The hydrostatic pressure non-equilibrium between the two tubes drew the liquid downwards through the cold tube as the two-phase mixture was released upwards through the hot tube. The authors concluded that the pool boiling performance of the pumpless cooling loop was better than that using conventional thermosyphons, given that the loop eliminated the incipience excursion and augmented the velocity of the fluid entering the hot tube, resulting in a greater amount of vapor in the hot tube that ameliorated the thermal performance with increasing heat flux. The authors also studied the general impact of the boiling section gap width on the water and FC-72 CHF. The CHF values for both thermal fluids were insensitive to the gap width for large gaps, whereas in cases where the gap width was lower by nearly 3.6 mm, the FC-72 CHF was raised due to the enhanced fluid flow velocity. The opposite trends of the two coolants were interpreted by the authors as being the result of the generation of very small bubbles when using the FC-72 that could easily pass through very narrow gaps, while the water generates much larger bubbles that may obstruct considerably the liquid replenishment in narrow gaps. Also, the authors Cardoso et al. [162] studied the boiling process using an upper horizontal surface and reported that, for gap widths decreasing from 13 to 0.1 mm, the FC-72 and FC-87 nucleate boiling heat transfer capability was enhanced at imposed heat fluxes lower than 4.5 W/cm^2^ but deteriorated the heat transfer for subcooled boiling. The enhancement was attributed to the lateral departure of the larger vapor bubbles, which aid in the drawing of the cold liquid into the boiling gap, and to the pulsation of the bubbles before departure.

### 2.3. Compound Enhancement

There have been many efforts from researchers to experimentally implement the combined usage of different approaches for nucleate boiling heat transfer enhancement and to achieve an overall thermal performance better than those of the individual approaches when carried out separately. Generally, the development and implementation of hybrid enhancement techniques have shown notorious improvements in both nucleate boiling HTC and CHF. Also, these routes can conjugate two or more active enhancement techniques, as described in this work, and can also conjugate one active enhancement technique with one or more surface enhancement techniques at all scales. Also, these schemes may involve surface modification within the same enhancement scale or combining different length scales. Generally, the implementation of novel micro/nano fabrication techniques has considerably broadened the development of hybrid enhanced surfaces, which have shown notorious improvements in the nucleate boiling HTC and CHF values. Nonetheless, this kind of enhanced surface usually poses major challenges that require in-depth further studies to be overcome, namely, the fact that the heat transfer mechanisms at micro/nanoscale are not yet completely understood, and much of the published works regarding surface modification at the same scale levels do not include an improved understanding of the aging effects. Indeed, it has already been demonstrated in recent studies based on prolonged boiling experiments that the micro/nanoscale heating surface features may deteriorate or, even, completely lose their heat transfer-enhancing capability over time. The following sub-sections present an overview of the published works regarding the compound enhancement approach using active and passive techniques together.

#### 2.3.1. Wall Deformation and Fluid Vibration

The work described in Section 2.1.9 of the present work of review can be considered a hybrid or compound enhancement approach with the conjunction of two active pool boiling enhancement techniques: confinement with wall deformation and ultrasound application.

#### 2.3.2. Fluid Vibration and Surface Enhancement

The authors Boziuk et al. [163] examined the pool boiling process under the influence of an ultrasonic field and using a micro-channeled and flat heating surface. The experimental results indicated that for the micro-channeled surface, the superheat was reduced by nearly 7 °C in comparison to that for the flat surface, and the CHF improved by up to 30%. The researchers argued that the microchannels not only augmented the number of active nucleation sites but also facilitated fluid transport to these sites as well. The fluid flow reduced the drag over the growing vapor bubbles and, hence, reduced the minimum required wall superheat.

#### 2.3.3. Electric Field and Surface Enhancement

The researchers Quan et al. [164] investigated the pool boiling heat transfer capability on smooth/ribbed heating surfaces under the influence of an external electric field. The results indicated that the detachment of the bubbles generated in between the ribs was hindered, which resulted in a degradation of the heat transfer performance at high heat fluxes under the electric field effect. Additionally, in the experimental work carried out by the authors Liu et al. [165], the combined effect of the application of an electric field and microstructured enhanced surfaces on the FC-72 pool boiling process at subcooling degrees of 5, 15, 25, and 35 K was analyzed. The employed enhanced surfaces were structured with different pin-fin sizes, and their heat transfer capability was compared with that of a smooth heating surface. Considering the obtained results, the authors arrived at the following conclusions: (i) It was found that CHF enhancements between 15.2% and 22.5% of the smooth surface occurred because of the effect of the electric field at low and high heat fluxes, and the enhancements increased with increasing liquid subcooling. (ii) It was observed that the electric field had negligible influence on the nucleate boiling heat transfer performance, except in cases where high heat fluxes were imposed for the microstructured heating surfaces. For these surfaces, the action of the applied electric field on the CHF value was closely linked to the liquid subcooling degree and with the width of the superficial pin-fins. For heating surfaces with a fin width of 30 μm, the increase in CHF caused by the effect of the electric field was insensitive to the liquid subcooling. Nonetheless, for heating surfaces with a fin width of 50 μm, the contribution of the action of the electric field for the CHF enhancement decreased with increasing subcooling. (iii) The enhancement of the CHF provoked by the electric field influence was interpreted by the authors as being the result of the competition between the reduction in the Taylor length and the disruption of the existent vapor layer, which enhances the CHF and the field trap effect that, in turn, deteriorates the CHF. (iv) The field trap effect on the boiling surface with a fin width of 50 μm became very strong at T_sub_ = 25 k and at T_sub_ = 35 K diminishing the CHF, which demonstrates that the electric field effect did not improved the CHF value in all the circumstances and that the higher liquid subcoolings strengthened the field trap effect, thereby further weakening the promoting influence of the electric field on the CHF.

#### 2.3.4. Local Jet Impingement and Surface Enhancement

Finned surfaces and structures combined with the two-phase local jet impingement have already been explored for the heat transfer enhancement of the pool boiling-assisted process [166]. The rationale behind the use of enhanced surfaces is to increase the available heat transfer area while enhancing the HTC through turbulent fluid transport and stabilization of the boundary layer. The single-phase results of the jet impingement action on enhanced surfaces have shown considerable HTC enhancements when compared to those obtained with smooth surfaces. For instance, an enhanced HTC value as high as nearly 150,000 W/m^2^ K was already reported for the R134a pool boiling process [61]. The cooling performance improvement of a single impinging jet using the HFE-7100 refrigerant and diverse enhanced surfaces was evaluated in the experimental work conducted by the authors Rau and Garimella [166]. In their work, the thermal performance of a 3.75 mm diameter jet was inferred on different copper surfaces that can be described as follows: a smooth surface, a surface coated with a microporous layer, an enhanced surface based on extended square pin fins, and a hybrid enhanced surface with square pin fins coated with a microporous layer. The nucleate boiling heat transfer enhancement and pressure drop provided by each experimented surface were compared to those of the smooth surface in single- and two-phase operation conditions. The imposed flow rates were 450, 900, and 1800 mL/min. The obtained experimental results revealed the following: (i) The pressure drop for all the experimented surfaces was independent of the imposed heat flux and vapor generation, and both enhanced surfaces did not affect the pressure drop significantly. (ii) The coated heating surface provided only a minor enhancement in the single-phase boiling heat transfer under higher jet velocities, which was interpreted by the authors as being the result of the increased turbulence in the wall-jet boundary layer. Also, the coated surface did not lead to any increase in the conduction thermal resistance compared to what happened with the comparative smooth surface, and displayed low wall superheats, CHF enhancements, and a large incipience overshoot. (iii) The enhanced boiling surfaces having coated and uncoated square pin fins provided a nearly 3-fold extension of the available surface heat transfer area and an appreciable enhancement in the HTC value. In the two-phase regime, the boiling surface modification with square pin fins reduced the temperature overshoot, decreased the nucleate boiling wall superheat between 6 and 8 °C, and enhanced the CHF. The experimental boiling curves for both pin-finned enhanced surfaces exhibited a reduced slope at high heat fluxes, which was attributed by the authors to the dry-out at the base of the pin fins. The minimum efficiencies of 0.8 and 0.7 for the uncoated and coated pin fin enhanced surfaces, respectively, highlight the gains in the nucleate boiling heat transfer capability that can be achieved with additional surface enhancement. (iv) The hybrid coated pin-finned surface presented the best performance of all the heating surfaces used in the experiments since it exhibited low incipience overshoot, low nucleate boiling wall superheats, and the highest CHF of all surfaces at all flow rates.

## 3. Influence of the Liquid Pool Height on the Boiling Heat Transfer Enhancement

Regardless of the pool boiling heat transfer enhancement route followed, the liquid pool height is an influencing factor that should be seriously considered. Since the removal of the vapor bubbles from the heating surface is critical to achieving a nucleate pool boiling enhanced heat transfer performance, it was hypothesized that if a bubble can reach rapidly the liquid–vapor interface, a gain in the pool boiling heat transfer performance can be achieved. Because of the movement of the bubbles in the liquid bulk, it becomes critical to understand the influence of the liquid height on the pool boiling heat transfer behavior. A major part of the published studies was performed with a liquid height appreciably greater than the vapor bubble diameter at the detachment from the surface stage. At lower liquid heights, the nucleating bubbles may interact with the free surface, leading to alterations in bubble growth and vapor formation. Hence, it has been clearly identified that there is a pressing need to evaluate the pool boiling heat transfer capability at liquid heights that are comparable to the bubble diameter at departure. Only a few authors have examined the pool boiling heat transfer performance at low liquid heights and heat fluxes lower than 10% of the CHF value. Apart from this, the authors Nishikawa et al. [167] studied the effect of the liquid height between 1 and 30 mm at imposed heat fluxes lower than 10 W/cm^2^ using water, ethyl alcohol, and an aqueous solution of sodium oleate as working fluids. The authors confirmed that as the liquid height was reduced from 30 to 5 mm, the nucleate HTC at a fixed heat flux remained unchanged, and with the further reduction in the operating fluid height below 5 mm, the nucleate HTC was found to rapidly increase. Additionally, in the study performed by the researchers Shukla and Kandlikar [168], the impact of the liquid pool height on the pool boiling heat transfer capability was evaluated at higher imposed heat fluxes. For this purpose, pool boiling experiments were conducted with water having a liquid height ranging from 2 mm to 50 mm over a copper boiling surface. In view of the experimental results, the authors deduced the following conclusions: (i) The CHF increased with increasing liquid height, but beyond 20 mm the CHF remained fixed at 125 W/cm^2^. (ii) At an imposed heat flux of 36 W/cm^2^ there were confirmed various vapor bubble formation mechanisms and the non-dimensional parameter H∗, which is the liquid height-to-departure bubble diameter ratio. The latter was found to be correlated with the vapor venting mechanisms impacting the heat transfer performance: when the ratio H∗ was lower than 4, the vapor venting mechanism observed at lower liquid height came into play, and in cases where H∗ was superior to 4, the mechanism observed at higher liquid height became notable. In cases where H∗ is superior to 4, the bubble detachment contributed to an enhanced CHF. (iii) It was experimentally confirmed that the bubble motion became more effortless with low liquid heights, resulting in a higher nucleate HTC value. Besides that, higher liquid heights offered enhanced rewetting of the boiling surface, resulting in a greater CHF value. (iv) The bubble detachment from the heating surface, bubble coalescence, and liquid height were responsible for the vapor venting and rewetting of the boiling surface through the liquid replenishment. It was hypothesized that when the liquid height and diameter of the bubbles at the departure stage become comparable, the changes in the vapor venting may ameliorate the nucleate HTC and that the liquid replenishment and CHF may be conditioned by the absence of bulk liquid above the nucleating bubbles.

## 4. Limitations, Challenges, and Recommendations for Further Research

The general recommendations and guidelines for further research topics concerning the challenging pool boiling enhancement field area of actuation can be summarized in the following points:

(i) Despite the verified progress in the nucleate pool boiling heat transfer performance employing innovative coating materials and techniques, the large-scale applications of these concepts remain hindered or, in some cases, even totally unpractical. To overcome such limitations, emphasis should be given to the durability and cost-effectiveness of the coatings, which are the main factors that still limit their large-scale implementation in applications like water harvesting, self-cleaning, aerospace, and thermal management based on the use of heat exchangers, heat tubes, thermosyphons, and other devices. In this direction, innovative fabrication methodologies are required to be implemented to ensure the cost-effectiveness, reliability, and longevity of the enhanced boiling surfaces. Although there have already been reports of effective enhancing methodologies, there is still a lack of comprehensive techno-economic analysis for these enhanced surfaces to be applied in practical nucleate boiling heat transfer situations.
(ii) Durability tests should be implemented on the coatings for performance inferring and to make a regular comparison of the employed methodologies and materials, allowing for further lifespan determination for the coatings. In addition, future studies should be conducted based on the analysis of the corrosion and erosion resistances, and adhesion strengths of the enhanced surfaces to be applied to nucleate pool boiling heat transfer enhancement ends.
(iii) The compound or hybrid enhancement route should be further investigated to better understand the possibilities of combining more than one enhancement technique searching for enhanced heat transfer capability, such as the inclusion of nanoparticles on microstructured porous coatings, the inclusion of nanotubes on microchannels, and the combined usage of microporous and hydrophilic or hydrophobic coatings.
(iv) Limiting values for CHF should be established using each type of enhanced heat transfer boiling surface, which should be achieved together with the durability testing results. Values for the CHF limits should be obtained for newly produced surfaces and for aged surfaces according to a well-defined start-up/cool-down cycle.
(v) A standard definition for a smooth heating surface should be adopted, which should be taken as the reference boiling surface in each heat transfer enhancement route, and its preparation method should be unified to properly address the enhancement level in the HTC and CHF and the respective inter-laboratorial comparison.
(vi) A ranking criterion should be assumed for structured surface enhancements in the nucleate HTC and CHF values under pool boiling scenarios. A minimum percentage compared to a bare, plain heating surface should be assumed, in which the enhancement qualifies the surface as possessing superior nucleate HTC and CHF. It would also be useful to classify the heat transfer enhancement techniques according to the experimental obtained results obtained by standardized methods under the same operating conditions.
(vii) The pool boiling regime offers a harsh environment for heat transfer surfaces. The existing high surface temperatures, together with the large interfacial fluid flow velocities, are adverse to the durability of surface features. Hence, the durability testing for each new enhanced structured surface should be performed with intermittent start-up and shut-down cycles. For instance, at least 10 start-up/shutdown cycles should be conducted, with each cycle lasting a few hours, followed by a cool-down for 24 h.
(viii) The published results often demonstrate the beneficial features of biphilic heating surfaces over conventional hydrophilic and hydrophobic ones. Nonetheless, there are still many aspects closely linked to this technological area that should be better known. For example, the sustainability of the hydrophobic regions should be investigated by calculating the heating wall shear stress to assess whether such surfaces can withstand the continuous bubble generation and detachment from the surface over time. Also, the shape, size, and pitch of the wettability patterns need to be optimized for heat transfer enhancement purposes.
(ix) The criteria for inferring CHF should be unified among the research community because it is clearly subjective. Most investigators detect CHF from the sudden rise of the heat transfer surface temperature but do not agree on its value. For instance, the authors Ahn et al. [169] recorded the CHF at a very high 86 K superheat, and the authors Kong et al. [170] recorded the CHF at only an 8.2 K superheat. This lack of uniformity in the experimental procedures will lead to a wider variation in the reliability of the actual CHF predictive equations and models and an inaccurate comparison of the nucleate pool boiling heat transfer enhancement results from different elements of the research community.

## 5. Conclusions

The present review provides a comprehensive survey of the most relevant studies addressing the active enhancement techniques of nucleate pool boiling heat transfer. The reviewed pool boiling heat transfer enhancing techniques included the main active techniques like the application of external fields, boiling confinement, and others. The current work also discussed the potential limitations and challenges closely linked to the implementation of heat transfer enhancement methodologies in thermal management applications. The main findings of this work can be summarized as follows:

(i) Pool boiling enhancement techniques usually achieve higher HTC and CHF values when compared with those provided through nucleate pool boiling alone. The most suitable pool boiling enhancement techniques should be selected considering the boiling space, the achievable nucleate boiling heat transfer performance, the investment cost, geometrical factors like the size and shape of the boiling surface, and the application field. The implementation of structured enhanced surfaces under pool boiling scenarios involves concerns intertwined from many aspects. On the one hand, the structures introduce surface irregularities and increase the number of active nucleation sites on the heating surface, which may improve the heat transfer performance. On the other hand, based on the Cassie–Baxter theory, the structures alter the contact angle and wettability, which control the interfacial hydrodynamic behavior of the vapor bubbles, promoting the initiation of the CHF. The pool boiling HTC and CHF increase with a nanostructured surface compared with an untreated surface. This increase can be appreciable, up to 100% or even more, depending on the material, thickness, and structure of the coating. Nonetheless, despite the large number of published works addressing pool boiling heat transfer enhancement, there is still a noticeable scarcity of sufficiently large available databases of surface enhancement methods regarding the fluid type, material, dimensions, and orientation of the heating surface as well as the enhancement methodology pattern and operating pressure. This evidence has led to less-than-adequate available findings for the design of enhanced surfaces for thermal management purposes.
(ii) The coating of the heating surface with nanoparticles, nanotubes, nanowires, nanofibers, nanoporous, and nanofilm layers, among other forms, has the advantage of nucleate boiling heat transfer enhancement by means of capillary wicking within the nanostructures. The taller nanotubes often give better nucleate HTC and CHF values. The underlying mechanisms for such results are not yet completely understood, but the availability of vapor embryos at low heat fluxes and pathways for liquid flow appears to be one of the fundamental reasons. Nonetheless, the topography of these nanostructures may lead to localized flow obstruction, hindering in any long-term enhancement. The coating of the boiling surfaces is mostly based on chemical synthesis or self-assembly, which is easy, scalable, cost-effective, and can be applied with different metals and metal oxides. However, the broadened distribution of possible shapes, sizes, and spacings in the nanotubes and nanoparticles is still challenging. Additionally, the interfacial strength between the nanoparticles and the substrate may not be sufficient to sustain the harsh environment provoked by the fluid or bubbles at high temperature under pool boiling conditions, causing film delamination, particle damage, or even detachment from the surface. The surface roughening and the incorporation of fins, microchannels, tunnels, reentrant cavities, and microporous structures, among others, have their main beneficial feature in the increased active nucleation site density that increases the nucleation boiling HTC. Also, the use of microporous structures is particularly noteworthy given their capability to enhance CHF values through the separation of the vapor and fluid pathways. The use of extended surfaces, porous mesh, and foam often provide different enhancements in the nucleate HTC and CHF values, together with the elimination of the incipience superheat. Also, the combined usage of extended surfaces with fluid subcooling is particularly effective for achieving nucleate boiling heat transfer benefits. Nonetheless, the attachment of an extended surface to a temperature-sensitive device may result in a contact resistance that will increase the device temperature, which may induce strong thermal stresses. Moreover, it can be concluded that the separation of the vapor and liquid pathways can be achieved by different procedures, for example, the following ones: (i) Mixed wettability, which enhances the liquid supply to the surface and thus increasing the CHF. This can be achieved by coating a hydrophilic surface with hydrophobic spots, where the bubbles nucleate and prevent the nucleation from the surrounding hydrophilic areas caused by the localized lateral cooling induced by the nucleation at the hydrophobic spots. The triple contact line does not spread beyond the hydrophobic spots, and thus lateral bubble coalescence diminishes, which creates space for the liquid to replenish the heating surface. (ii) The heating surface is coated with a porous layer over which pin fins are created. The bubbles nucleate from the flat porous areas, whereas liquid replenishment occurs through the vertical porous by capillary wicking. The bubbles nucleate over the pin fins, and the capillary liquid supply occurs through the non-finned areas. (iii) Using parallel microchannels with coated fins, the vapor bubbles will nucleate from the porous fin tip, and the liquid falls and impinges on the channel bottom, resulting in heat transfer enhancement.
(iii) The compound or hybrid enhancement techniques include the use of one active technique together with a passive technique and two or more active techniques, like the combined usage of space confinement wall deformation and ultrasound waves. These combined techniques often exhibit promising nucleate pool boiling heat transfer enhancement trends. Considering safe CHF enhancement as a factor of chief importance, the integration of advanced approaches, such as ultrasound-assisted methods, magnetic fields, and other techniques, through innovative preparation methods and materials is highly recommended. Nonetheless, the hybrid surface enhancements that involve the use of nanocoatings are susceptible to obstruction and deterioration over time. Also, the integration of hybrid and hierarchical structures makes the accurate evaluation of the impact due to the isolated role of the surface features on CHF amelioration difficult.
(iv) The selection of the fabrication process plays a relevant role according to cost, durability, reliability, and effectiveness standpoints. For instance, lithography can define surface geometric features and spacing with very high resolution. Such features allow the study of the impact of the surface geometric features on the boiling phenomena. Additionally, lithography is compatible with the silicon IC/MEMS processes, allowing their applications to be integrated with silicon devices. On the other hand, techniques using nanoporous materials, which can result in nanostructures with controllable dimensions and density, are found to be cost-effective and scalable. All the enhanced surface fabrication methodologies offer a suitable strategy to produce structured surfaces and control their wettability, but all of them also have their beneficial and disadvantageous features regarding geometry; size; density; and uniformity of the surface nanostructures, equipment, material reliability and durability, and boiling enhanced heat transfer application. Also, the selection of the material of the surface structures is relevant because they must present enough robustness to endure high temperatures and harsh environments during the pool boiling process. Considering the cost, thermophysical properties, and manufacturability, the choice of the most suitable material should be made. Silicon has the highest material cost per unit area, but it is often selected due to its excellent mechanical strength and thermal conductivity. The metals also possess superior mechanical strength and thermophysical properties, which allow them to be applied for many boiling heat transfer purposes.

## Data Availability

Data sharing is not applicable.

## References

[1] Kerlin T.W., Upadhyaya B.R. (2019). Chapter 13—Boiling water reactors. Dynamics and Control of Nuclear Reactors.

[2] Singh T., Atieh M.A., Al-Ansari T., Mohammad A.W., Mckay G. (2020). The Role of Nanofluids and Renewable Energy in the Development of Sustainable Desalination Systems: A Review. Water.

[3] Moita A., Moreira A., Pereira J. (2021). Nanofluids for the Next Generation Thermal Management of Electronics: A Review. Symmetry.

[4] Sureshkumar R., Mohideen S.T., Nethaji N. (2013). Heat transfer characteristics of nanofluids in heat pipes: A review. Renew. Sustain. Energy Rev..

[5] Dixit T., Ghosh I. (2015). Review of micro- and mini-channel heat sinks and heat exchangers for single phase fluids. Renew. Sustain. Energy Rev..

[6] Zhang K.-I., Liu Z.-H., Zheng B.-C. (2016). A new 3D chip cooling technology using micro-channels thermosyphon with super-moist fluids and nanofluids. Energy Convers. Manag..

[7] Bergman T.L., Incropera F.P., DeWitt D.P., Lavine A.S. (2011). Fundamentals of Heat and Mass Transfer.

[8] Khan S.A., Atieh M.A., Koç M. (2018). Micro-Nano Scale Surface Coating for Nucleate Boiling Heat Transfer: A Critical Review. Energies.

[9] Hsu C.-C., Chen P.-H. (2012). Surface wettability effects on critical heat flux of boiling heat transfer using nanoparticle coatings. Int. J. Heat Mass Transf..

[10] Motezakker A.R., Sadaghiani A.K., Çelik S., Larsen T., Villanueva L.G., Kosar A. (2019). Optimum ratio of hydrophobic to hydrophilic areas of biphilic surfaces in thermal fluid systems involving boiling. Int. J. Heat Mass Transf..

[11] Shen B., Mine T., Iwata N., Hidaka S., Takahashi K., Takata Y. (2020). Deterioration of boiling heat transfer on biphilic surfaces under very low pressures. Exp. Therm. Fluid Sci..

[12] Kim D.E., Yu D.I., Jerng D.W., Kim M.H., Ahn H.S. (2015). Review of boiling heat transfer enhancement on micro/nanostructured surfaces. Exp. Therm. Fluid Sci..

[13] Kalita S., Sen P., Sen D., Das S., Das A.K., Saha B.B. (2021). Experimental study of nucleate pool boiling heat transfer on microporous structured by chemical etching method. Therm. Sci. Eng. Prog..

[14] Mudhafar M.A.H., Zheng-hao W. (2022). Optimization of Pool Boiling Heat Transfer on microporous metal coating surfaces with FC-72 as a working fluid. Heat Mass Transf..

[15] Moghadasi H., Malekian N., Saffari H., Gheitaghy A.M., Zhang G.Q. (2020). Recent Advances in the Critical Heat Flux Amelioration of Pool Boiling Surfaces Using Metal Oxide Nanoparticle Deposition. Energies.

[16] Zhao H., Dash S., Dhillon N.S., Kim S., Lettiere B., Varanasi K.K., Hart A.J. (2019). Microstructured Ceramic-Coated Carbon Nanotube Surfaces for High Heat Flux Pool Boiling. ACS Appl. Nano Mater..

[17] Lee S., Seo G.H., Lee S., Jeong U., Lee S.J., Kim S.J., Choi W. (2016). Layer-by-layer carbon nanotube coatings for enhanced pool boiling heat transfer on metal surfaces. Carbon.

[18] Mao L., Zhou W., Hu X., He Y., Zhang G., Zhang L., Fu R. (2020). Pool boiling performance and bubble dynamics on graphene oxide nanocoating surface. Int. J. Therm. Sci..

[19] Liang G., Mudawar I. (2019). Review of pool boiling enhancement by surface modification. Int. J. Heat Mass Transf..

[20] Cho H.J., Wang E.N. (2019). Bubble nucleation, growth, and departure: A new, dynamic understanding. Int. J. Heat Mass Transf..

[21] Liu J., Orejon D., Zhang N., Terry J.G., Walton A.J., Sefiane K. (2024). Bubble Coalescence During Pool Boiling with Different Surface Characteristics. Heat Transf. Eng..

[22] Wang X., Fadda D., Godinez J., Lee J., You S.M. (2022). Effect of wettability on pool boiling heat transfer with copper microporous coated surface. Int. J. Heat Mass Transf..

[23] Xu N., Liu Z., Yu X., Gao J., Chu H. (2024). Processes, models and the influencing factors for enhanced boiling heat transfer in porous structures. Renew. Sustain. Energy Rev..

[24] Bergles A.E. (1969). The Influence of Heated-Surface Vibration on Pool Boiling. J. Heat Transfer..

[25] Hosseinian A., Isfahani A.H.M., Shirani E. (2018). Experimental investigation of surface vibration effects on increasing the stability and heat transfer coefficient of MWCNTs-water nanofluid in a flexible double pipe heat exchanger. Exp. Therm. Fluid Sci..

[26] Unno N., Yuki K., Taniguchi J., Satake S. (2020). Boiling heat transfer enhancement by self-excited vibration. Int. J. Heat Mass Transf..

[27] Zhang B., Gong S., Dong S., Xiong Z., Guo Q. (2023). Experimental investigation on vibration characteristics of subcooled and saturated pool boiling. Appl. Therm. Eng..

[28] Prisnyakov V.F., Navruzov Y.V., Mamotov P.V., Stoichev A.V. (1992). Characteristics of heat emission from a vibrating heat source in a vessel with liquid. Teplofiz. Vysok. Temp..

[29] Zitko V., Afgan N. (1994). Boiling Heat Transfer from Oscillating Surface. J. Enhanc. Heat Transf..

[30] Atashi H., Alaei A., Kafshgari M.H., Aeinehvand R., Rahimi S.K. (2014). New Pool Boiling Heat Transfer in the Presence of Low-Frequency Vibrations Into a Vertical Cylindrical Heat Source. Exp. Heat Transf..

[31] Sathyabhama A., Prashanth S.P. (2017). Enhancement of Boiling Heat Transfer Using Surface Vibration. Heat Transf..

[32] Abadi S.M.A.N.R., Ahmadpour A., Meyer J.P. (2019). Effects of vibration on pool boiling heat transfer from a vertically aligned array of heated tubes. Int. J. Multiph. Flow.

[33] Alangar S. (2017). Effect of boiling surface vibration on heat transfer. Heat Mass Transf..

[34] Alimoradi H., Zaboli S., Shams M. (2022). Numerical simulation of surface vibration effects on improvement of pool boiling heat transfer characteristics of nanofluid. Korean J. Chem. Eng..

[35] Brennen C.E. (2014). Cavitation and Bubble Dynamics.

[36] Quintana-Buil G., González-Cinca R. (2021). Acoustic effects on heat transfer on the ground and in microgravity conditions. Int. J. Heat Mass Transf..

[37] Baffigi F., Bartoli C. (2009). Heat transfer enhancement from a circular cylinder to distilled water by ultrasonic waves in subcooled boiling conditions. Interdisciplinary Transport Phenomena VI: Fluid, Thermal, Biological, Materials and Space Sciences.

[38] Tang J., Sun L., Wu D., Du M., Xie G., Yang K. (2019). Effects of ultrasonic waves on subcooled pool boiling on a small plain heating surface. Chem. Eng. Sci..

[39] Bartoli C., Baffigi F. (2012). Use of ultrasonic waves in sub-cooled boiling. Appl. Therm. Eng..

[40] Legay M., Gondrexon N., Le Person S., Boldo P., Bontemps A. (2011). Enhancement of Heat Transfer by Ultrasound: Review and Recent Advances. Int. J. Chem. Eng..

[41] Hetsroni G., Moldavsky L., Fichman M., Pogrebnyak E., Mosyak A. (2014). Ultrasonic enhancement of subcooled pool boiling of freely oscillated wires. Int. J. Multiph. Flow.

[42] Sitter J.S., Snyder T.J., Chung J.N., Marston P.L. (1998). Acoustic field interaction with a boiling system under terrestrial gravity and microgravity. J. Acoust. Soc. Am..

[43] Kim H.-Y., Kim Y.G., Kang B.H. (2004). Enhancement of natural convection and pool boiling heat transfer via ultrasonic vibration. Int. J. Heat Mass Transf..

[44] Mondal K., Bhattacharya A. (2021). Pool boiling enhancement through induced vibrations in the liquid pool due to moving solid bodies—A numerical study using the lattice Boltzmann method (LBM). Phys. Fluids.

[45] Suriyawong A., Saisorn S., Wongwises S. (2017). Pool boiling heat transfer enhancement of distilled water with passive rotating blades installed above the heating surface. Exp. Therm. Fluid Sci..

[46] Ashouri M., Rahmati P., Hakkaki-Fard A. (2022). On the effect of corrugated conical frustum on pool boiling heat transfer. Exp. Therm. Fluid Sci..

[47] Zaghdoudi C., Lallemand M. (2005). Pool Boiling Heat Transfer Enhancement by Means of High DC Electric Field. Arab. J. Sci. Eng..

[48] Hristov Y., Zhao D., Kenning D.B.R., Sefiane K., Karayiannis T.G. (2009). A study of nucleate boiling and critical heat flux with EHD enhancement. Heat Mass Transf..

[49] Di Marco P., Grassi W. (2011). Effects of external electric field on pool boiling: Comparison of terrestrial and microgravity data in the ARIEL experiment. Exp. Therm. Fluid Sci..

[50] Darabi J., Ekula K. (2003). Development of a chip-integrated micro cooling device. Microelectron. J..

[51] Nguyen T. Electro-Hydro-Dynamics- Enhancement of Multi-Phase Heat Transfer, Faculty of Engineering (Mechanical), University of Technology, Sydney, Australia. https://www.slideserve.com/lucky/electro-hydro-dynamics-enhancement-of-multi-phase-heat-transfer-powerpoint-ppt-presentation.

[52] Ozdemir M.R., Sadaghiani A.K., Motezakker A.R., Parapari S.S., Park H.S., Acar H.Y., Kosar A. (2018). Experimental studies on ferrofluid pool boiling in the presence of external magnetic force. Appl. Therm. Eng..

[53] Rhamati P., Ebrahimi-Dehshali M., Hakkaki-Fard A. (2020). Enhancement of pool boiling heat transfer using ferromagnetic beads in a variable magnetic field. Appl. Therm. Eng..

[54] O’Connor J.P., You S.M., Chang J.Y. (1996). Gas-Saturated Pool Boiling Heat Transfer from Smooth and Microporous Surfaces in FC-72. J. Heat Transf..

[55] You S.M., Simon T.W., Bar-Cohen A. (1990). Experiments on nucleate boiling heat transfer with a highly-wetting dielectric fluid. Effects of Pressure, Subcooling and Dissolved Gas Content.

[56] Sarafraz M.M., Peyghambarzadeh S.M., Fazel S.A.A. (2012). Enhancement of the pool boiling heat transfer coefficient using the gas injection into the water. Pol. J. Chem. Technol..

[57] Zhang Y., Liu W., Liu B., Yu X., Wei J. (2021). Experimental Study of Enhanced Boiling Heat Transfer with Suction. Microgravity Sci. Technol..

[58] Mitsutake Y., Monde M. (2003). Ultra High Critical Heat Flux During Forced Flow Boiling Heat Transfer with an Impingement Jet. ASME J. Heat Transf..

[59] Ma C.-F., Bergles A.E. (1986). Jet impingement nucleate boiling. Int. J. Heat Mass Transf..

[60] Wolf D.H., Incropera F.P., Viskanta R. (1996). Local jet impingement boiling heat transfer. Int. J. Heat Mass Transf..

[61] Ndao S., Peles Y., Jensen M.K. (2012). Experimental investigation of flow boiling heat transfer of jet impingement on smooth and micro structured surfaces. Int. J. Heat Mass Transf..

[62] Leal L., Lavieille P., Miscevic M., Pigache F., Tadrist L. Control of pool boiling incipience in confined space: Dynamic morphing of the wall effect. Proceedings of the 3rd Micro and Nano Flows Conference.

[63] Jun S., Kim J., You S.M., Kim H.Y. (2016). Effect of heater orientation on pool boiling heat transfer from sintered copper microporous coating in saturated water. Int. J. Heat Mass Transf..

[64] An S., Kim D.-Y., Lee J.-G., Jo H.S., Kim M.-W., Al-Deyab S.S., Choi J., Yoon S.S. (2016). Supersonically sprayed reduced graphene oxide film to enhance critical heat flux in pool boiling. Int. J. Heat Mass Transf..

[65] Ho J.Y., Wong K.K., Leong K.C., Yang C. Enhanced Nucleate Pool Boiling from Microstructured Surfaces Fabricated by Selective Laser Melting. Proceedings of the ASME 2016 5th International Conference on Micro/Nanoscale Heat and Mass Transfer, Biopolis.

[66] Chen S.-W., Hsieh J.-C., Chou C.-T., Lin H.-H., Shen S.-C., Tsai M.-J. (2007). Experimental investigation and visualization on capillary and boiling limits of micro-grooves made by different processes. Sens. Actuators A Phys..

[67] Koizumi Y., Ohtake H., Sato T. Pool Boiling Characteristics of Heat Transfer Surface with Micro Structures Created by Using MEMS Technology. Proceedings of the 14th International Heat Transfer Conference.

[68] Watanabe Y., Enoki K., Okawa T. (2018). Nanoparticle layer detachment and its influence on the heat transfer characteristics in saturated pool boiling of nanofluids. Int. J. Heat Mass Transf..

[69] Wu W., Bostanci H., Chow L.C., Hong Y., Su M., Kizito J.P. (2010). Nucleate boiling heat transfer enhancement for water and FC-72 on titanium oxide and silicon oxide surfaces. Int. J. Heat Mass Transf..

[70] Forrest E., Williamson E., Buongiorno J., Hu L.-W., Rubner M., Cohen R. (2010). Augmentation of nucleate boiling heat transfer and critical heat flux using nanoparticle thin-film coatings. Int. J. Heat Mass Transf..

[71] Chen M.-H., Chuang Y.-J., Tseng F.-G. (2008). Self-masked high-aspect-ratio polymer nanopillars. Nanotechnology.

[72] White S.B., Shih A.J., Pipe K.P. (2011). Boiling surface enhancement by electrophoretic deposition of particles from a nanofluid. Int. J. Heat Mass Transf..

[73] Yeom H., Sridharan K., Corradini M. (2015). Bubble Dynamics in Pool Boiling on Nanoparticle-coated Surfaces. Heat Transf. Eng..

[74] Souza R.R., Gonçalves I.M., Rodrigues R.O., Minas G., Miranda J.M., Moreira A.L.N., Lima R., Coutinho G., Pereira J.E., Moita A.S. (2022). Recent advances on the thermal properties and applications of nanofluids: From nanomedicine to renewable energies. Appl. Therm. Eng..

[75] Freitas E., Pontes P., Cautela R., Bahadur V., Miranda J., Ribeiro A.P.C., Souza R.R., Oliveira J.D., Copetti J.B., Lima R. (2021). Pool Boiling of Nanofluids on Biphilic Surfaces: An Experimental and Numerical Study. Nanomaterials.

[76] Kiyomura I.S., Manetti L.L., Cunha A.P., Ribatski G., Cardoso E.M. (2017). An analysis of the effects of nanoparticles deposition on characteristics of the heating surface and on pool boiling of water. Int. J. Heat Mass Transf..

[77] Souza R.R., Passos J.P., Cardoso E.M. (2014). Influence of nanoparticle size and gap size on nucleate boiling using HFE7100. Exp. Therm. Fluid Sci..

[78] Shah K.A., Tali B.A. (2016). Synthesis of carbon nanotubes by catalytic chemical vapor deposition: A review on carbon sources, catalysts and substrates. Mater. Sci. Semicond. Process..

[79] Ahn H.S., Sinha N., Zhang M., Banerjee D., Fang S., Baughman R.H. (2006). Pool Boiling Experiments on Multiwalled Carbon Nanotube (MWCNT) Forests. J. Heat Transf..

[80] Ujereh S., Fisher T., Mudawar I. (2007). Effects of carbon nanotube arrays on nucleate pool boiling. Int. J. Heat Mass Transf..

[81] Jaikumar A., Gupta A., Kandlikar S.G., Yang C.-Y., Su C.-Y. (2007). Scale effects of graphene and graphene oxide coatings on pool boiling enhancement mechanisms. Int. J. Heat Mass Transf..

[82] Kumar G.U., Soni K., Suresh S., Ghosh K., Thansekhar M.R., Babu P.D. (2018). Modified surfaces using seamless graphene/carbon nanotubes based nanostructures for enhancing pool boiling heat transfer. Exp. Therm. Fluid Sci..

[83] Wang W., Varghese O.K., Paulose M., Grimes G.A., Wang Q., Dickey E.C. (2004). A study on the growth and structure of titania nanotubes. J. Mater. Res..

[84] Chen Y., Mo D., Zhao H., Ding N. (2009). Pool boiling on the superhydrophilic surface with TiO_2_ nanotube arrays. Sci. China Ser. E Technol. Sci..

[85] Yao Z., Lu Y.-W., Kandlikar S.G. (2011). Direct growth of copper nanowires on a substrate for boiling application. Micro Nano Lett..

[86] Kumar G.U., Suresh S., Thansekhar M.R., Babu P.D. (2017). Effect of diameter of metal nanowires on pool boiling heat transfer with FC-72. Appl. Surf. Sci..

[87] Wen R., Li Q., Wang W., Latour B., Li C.H., Li C., Lee Y.-C., Yang R. (2017). Enhanced bubble nucleation and liquid rewetting for highly efficient boiling heat transfer on two-level hierarchical surfaces with patterned copper nanowire arrays. Nano Energy.

[88] Ray M., Deb S., Bhaumik S. (2016). Pool boiling heat transfer of refrigerant R-134a on TiO_2_ nano wire arrays surface. Appl. Therm. Eng..

[89] Jun S., Ray S.S., Yarin A.L. (2013). Pool boiling on nano-textured surfaces. Int. J. Heat Mass Transf..

[90] Choi C.-H., Krishnan S., TeGrotenhuis W., Chang C.-H. (2018). Capillary Rise of Nanostructured Microwicks. Micromachines.

[91] Mahmoud M.M., Karayiannis T.G. (2021). Pool boiling review: Part II—Heat transfer enhancement. Therm. Sci. Eng. Prog..

[92] Patil C.M., Kandlikar S.G. (2014). Review of the manufacturing techniques for porous surfaces used in enhanced pool boiling. Heat Transf. Eng..

[93] Jun S., Wi H., Gurung A., Amaya M., You S.M. (2016). Pool Boiling Heat Transfer Enhancement of Water Using Brazed Copper Microporous Coatings. J. Heat Transf..

[94] Seo H., Chu J.H., Kwon S.-Y., Bang I.C. (2015). Pool boiling CHF of reduced graphene oxide, graphene, and SiC-coated surfaces under highly wettable FC-72. Int. J. Heat Mass Transf..

[95] Sarangi S., Weibel J.A., Garimella S.V. (2017). Quantitative Evaluation of the Dependence of Pool Boiling Heat Transfer Enhancement on Sintered Particle Coating Characteristics. J. Heat Transf..

[96] Nishikawa K., Ito T. (1980). Augmentation of nucleate boiling heat transfer by prepared surfaces. Heat Transfer in Energy Problems.

[97] O’Neill P.S., Gottzmann C.F., Terbot J.W. (1972). Novel Heat Exchangers Increases Cascade Cycle Efficiency for Natural Gas Liquefaction. Advances in Cryogenic Engineering.

[98] Jaikumar A., Rishi A., Gupta A., Kandlikar S.G. (2017). Microscale morphology effects of copper-graphene oxide coatings on pool boiling characteristics. J. Heat Transf..

[99] Protich Z., Santhanam K.S.V., Jaikumar A., Kandlikar S.G., Wong P. (2016). Electrochemical Deposition of Copper in Graphene Quantum Dot Bath: Pool Boiling Enhancement. J. Electrochem. Soc..

[100] Li C., Peterson G.P. (2007). Parametric Study of Pool Boiling on Horizontal Highly Conductive Microporous Coated Surfaces. J. Heat Transf..

[101] Tang Y., Tang B., Li Q., Qing J. (2013). Pool-boiling enhancement by novel metallic nanoporous surface. Exp. Therm. Fluid Sci..

[102] Lu L., Fu T., Tang Y., Tang T., Tang B., Wan Z. (2016). A novel in-situ nanostructure forming route and its application in pool-boiling enhancement. Exp. Therm. Fluid Sci..

[103] Hendricks T.J., Krishnan S., Choi C., Chang C.-H. (2010). Enhancement of Pool Boiling Heat Transfer Using Nanostructured Surfaces on Aluminum and Copper. Int. J. Heat Mass Transf..

[104] Baig N., Kammakakam I., Falath W. (2021). Nanomaterials: A review of synthesis methods, properties, recent progress, and challenges. Mater. Adv..

[105] Nakayama W., Daikoku T., Kuwahara H., Nakajima T. (1980). Dynamic Model of Enhanced Boiling Heat Transfer on Porous Surfaces—Part I: Experimental Investigation. J. Heat Transf..

[106] Pastuzko R. (2012). Pool boiling for extended surfaces with narrow tunnels—Visualization and a simplified model. Exp. Therm. Fluid Sci..

[107] Halon T., Zajaczkowski B., Michaei S., Rulliere R., Bonjour J. (2017). Experimental study of low pressure pool boiling of water from narrow tunnel surfaces. Int. J. Therm. Sci..

[108] Moita A.S., Teodori E., Moreira A.L.N. (2015). Influence of surface topography in the boiling mechanisms. Int. J. Heat Fluid Flow.

[109] Teodori E., Moita A.S., Moreira A.L.N. Evaluation of pool boiling heat transfer over micro-structured surfaces by combining high-speed visualization and PIV measurements. Proceedings of the 10th International Symposium on Particle Image Velocimetry—PIV13.

[110] Ji W.-T., Zhao C.-Y., Zhang D.-C., Zhao P.-F., Li Z.-Y., He Y.-L., Tao W.-Q. (2017). Pool boiling heat transfer of R134a outside reentrant cavity tubes at higher heat flux. Appl. Therm. Eng..

[111] Bergles A.E. (2011). Augmentation of Heat Transfer, Two-Phase. Thermopedia—A-To-Z Guide to Thermodynamics, Heat & Mass Transfer, and Fluids Engineering.

[112] Hwang G.S., Fleming E., Carne B., Sharratt S., Nam Y., Dussinger P., Ju Y.S., Kaviany M. (2011). Multi-artery heat pipe spreader: Lateral liquid supply. Int. J. Heat Mass Transf..

[113] Nasersharifi Y., Kaviany M., Hwang G. (2018). Pool-boiling enhancement using multilevel modulated wick. Appl. Therm. Eng..

[114] Raghupathi P.A., Kandlikar S.G. (2017). Pool boiling enhancement through contact line augmentation. Appl. Phys. Lett..

[115] Deghani-Ashkezari E., Salimpour M.R. (2018). Effect of groove geometry on pool boiling heat transfer of water-titanium oxide nanofluid. Heat Mass Transf..

[116] Tang K., Bai J., Chen S., Zhang S., Li J., Sun Y., Chen G. (2021). Pool Boiling Performance of Multilayer Micromeshes for Commercial High-Power Cooling. Micromachines.

[117] Mudawar I., Anderson T.M. (1993). Optimization of Enhanced Surfaces for High Flux Chip Cooling by Pool Boiling. J. Electron. Packag..

[118] Rainey K.N., You S.M. (2000). Pool boiling heat transfer from plain and microporous square pin-finned surfaces in saturated FC-72. J. Heat Transf..

[119] Chang J.Y., You S.M. (1997). Enhanced Boiling Heat Transfer from Micro-Porous Cylindrical Surfaces in Saturated FC-87 and R-123. J. Heat Transf..

[120] Rioux R.P., Nolan E.C., Li C.H. (2014). A systematic study of pool boiling heat transfer on structured porous surfaces: From nanoscale through microscale to macroscale. AIP Adv..

[121] Li C.H., Rioux R.P. (2016). Independent and collective roles of surface structures at different length scales on pool boiling heat transfer. Sci. Rep..

[122] Chu K.-H., Joung Y.S., Enright R., Buie C.R., Wang E.N. (2013). Hierarchically structured surfaces for boiling critical heat flux enhancement. Appl. Phys. Lett..

[123] Rahman M.M., Olçeroglu E., McCarthy M. (2014). Role of Wickability on the Critical Heat Flux of Structured Superhydrophilic Surfaces. Langmuir.

[124] Zuber N. (1958). On the Stability of Boiling Heat Transfer. Trans. Am. Soc. Mech. Eng..

[125] Rahman M.M., McCarthy M. (2017). Effect of length scales on the boiling enhancement of structured copper surfaces. J. Heat Transf..

[126] Betz A.R., Jenkins J., Kim C.-J., Attinger D. (2013). Boiling heat transfer on superhydrophilic, superhydrophobic, and superbiphilic surfaces. Int. J. Heat Mass Transf..

[127] Phan H.T., Caney N., Marty P., Colasson S., Gavillet J. (2009). Surface wettability control by nanocoating: The effects on pool boiling heat transfer and nucleation mechanism. Int. J. Heat Mass Transf..

[128] Kim J.M., Yu D.I., Park H.S., Moriyama K., Kim M.H. (2017). Smart surface in pool boiling: Thermally-induced wetting transition. Int. J. Heat Mass Transf..

[129] Jo H.J., Kim S.H., Kim H., Kim J., Kim M.H. (2012). Nucleate boiling performance on nano/microstructures with different wetting surfaces. Nanoscale Res. Lett..

[130] Betz A.R., Xu J., Qiu H., Attinger D. (2010). Do surfaces with mixed hydrophilic and hydrophobic areas enhance pool boiling?. Appl. Phys. Lett..

[131] Choi C.-H., David M., Gao Z., Chang A., Allen M., Wang H., Chang C.-H. (2016). Large-scale Generation of Patterned Bubble Arrays on Printed Bi-functional Boiling Surfaces. Sci. Rep..

[132] Bertossi R., Caney N., Gruss J.A., Poncelet O. (2015). Pool boiling enhancement using switchable polymers coating. Appl. Therm. Eng..

[133] Azarkish H. (2020). Pool boiling enhancement on biphilic micropillar arrays: Control on the thin film evaporator and rewetting flow. Numer. Heat Transf. Part A Appl..

[134] Zhang J., Zou Z., Fu C. (2023). A Review of the Complex Flow and Heat Transfer Characteristics in Microchannels. Micromachines.

[135] Kim J., Jun S., Laksnarain R., You S.M. (2016). Effect of surface roughness on pool boiling heat transfer at a heated surface having moderate wettability. Int. J. Heat Mass Transf..

[136] Chaudhri I.H., McDougall I.R. (1969). Ageing studies in nucleate pool boiling of isopropyl acetate and perchloroethylene. Int. J. Heat Mass Transf..

[137] Kim J., Jun S., Lee J., Godinez J., You S.M. (2017). Effect of Surface Roughness on Pool Boiling Heat Transfer of Water on a Superhydrophilic Aluminum Surface. J. Heat Transf..

[138] Kim J.S., Girard A., Jun S., Lee J., You S.M. (2018). Effect of surface roughness on pool boiling heat transfer of water on hydrophobic surfaces. Int. J. Heat Mass Transf..

[139] Ferjancic K., Golobic I. (2002). Surface effects on pool boiling CHF. Exp. Therm. Fluid Sci..

[140] Fan S., Jiao L., Wang K., Duan F. (2020). Pool boiling heat transfer of saturated water on rough surfaces with the effect of roughening techniques. Int. J. Heat Mass Transf..

[141] Chu K.-H., Enright R., Wang E.N. (2012). Structured surfaces for enhanced pool boiling heat transfer. Appl. Phys. Lett..

[142] Kandlikar S.G. (2001). A Theoretical Model to Predict Pool Boiling CHF Incorporating Effects of Contact Angle and Orientation. J. Heat Transf..

[143] Kandlikar S.G. (2013). Controlling bubble motion over heated surface through evaporation momentum force to enhance pool boiling heat transfer. Appl. Phys. Lett..

[144] McNeil D.A., Raeisi A.H., Kew P.A., Hamed R.S. (2015). The effect of substrate conduction on boiling data on pin-fin heat sinks. Appl. Therm. Eng..

[145] Deng Z., Liu X., Wu S., Zhang C. (2021). Pool boiling heat transfer enhancement by bi-conductive surfaces. Int. J. Therm. Sci..

[146] Heidary A., Moghadasi H., Saffari H. (2021). Impact of dimensional characteristics of low-conductive channels on the enhancement of pool boiling: An experimental analysis. Int. J. Mech. Sci..

[147] Rahman M.M., Pollack J., McCarthy M. (2015). Increasing Boiling Heat Transfer using Low Conductivity Materials. Sci. Rep..

[148] Xu J., Ji X., Zhang W., Liu G. (2008). Pool boiling heat transfer of ultra-light copper foam with open cells. Int. J. Multiph. Flow.

[149] Manetti L.L., Moita A.S.O.H., Souza R.R., Cardoso E.M. (2020). Effect of copper foam thickness on pool boiling heat transfer of HFE-7100. Int. J. Heat Mass Transf..

[150] Yang Y., Ji X., Xu J. (2010). Pool boiling heat transfer on copper foam covers with water as working fluid. Int. J. Therm. Sci..

[151] Xu Z.G., Zhao C.Y. (2015). Experimental study on pool boiling heat transfer in gradient metal foams. Int. J. Heat Mass Transf..

[152] Shi J., Jia X., Feng D., Chen Z., Dang C. (2020). Wettability effect on pool boiling heat transfer using a multiscale copper foam surface. Int. J. Heat Mass Transf..

[153] Hayes A., Raghupathi P.A., Emery T.S., Kandlikar S.G. (2019). Regulating flow of vapor to enhance pool boiling. Appl. Therm. Eng..

[154] Chauhan A., Kandlikar S.G. (2019). Characterization of a Dual Taper Thermosiphon Loop for CPU Cooling in Data Centers. Appl. Therm. Eng..

[155] Chauhan A. (2021). High Heat Flux Dissipation Using Innovative Dual Tapered Manifold in Pool Boiling, and Thermosiphon Loop for CPU Cooling in Data Centers. Ph.D. Thesis.

[156] Chauhan A., Kandlikar S.G. (2022). Geometrical effects on heat transfer mechanisms during pool boiling in Dual Tapered Microgap with HFE7000. Int. J. Heat Mass Transf..

[157] Mokkapati V., Lin C.-H. (2019). Numerical Study of a Exhaust Heat Recovery System Using Corrugated Tube Heat Exchanger with Modified Twisted Tape Inserts. Int. J. Energy Eng..

[158] Nanan K., Thianpong C., Promvonge P., Eiamsa-Ard S. (2014). Investigation of heat transfer enhancement by perforated helical twisted-tapes. Int. Commun. Heat Mass Transf..

[159] Ebrahimi-Dehshali M., Najm-Barzanji S.Z., Hakkaki-Fard A. (2018). Pool boiling heat transfer enhancement by twisted-tape fins. Appl. Therm. Eng..

[160] Geisler K.J.L., Bar-Cohen A. (2009). Confinement effects on nucleate boiling and critical heat flux in buoyancy-driven microchannels. Int. J. Heat Mass Transf..

[161] Mukherjee S., Mudawar I. (2003). Smart Pumpless Loop fo Micro-Channel Electronic Cooling Using Flat and Enhanced Surfaces. IEEE Trans. Compon. Packag. Technol..

[162] Cardoso E.M., Kannengieser O., Stutz B., Passos J.C. (2011). FC72 and FC87 nucleate boiling inside a narrow horizontal space. Exp. Therm. Fluid Sci..

[163] Boziuk T.R., Smith M.K., Glezer A. (2017). Enhanced boiling heat transfer on plain and featured surfaces using acoustic actuation. Int. J. Heat Mass Transf..

[164] Quan X., Gao M., Cheng P., Li J. (2015). An experimental investigation of pool boiling heat transfer on smooth/rib surfaces under an electric field. Int. J. Heat Mass Transf..

[165] Liu B., Garivalis A.I., Cao Z., Zhang Y., Wei J., Di Marco P. (2022). Effects of electric field on pool boiling heat transfer over microstructured surfaces under different liquid subcoolings. Int. J. Heat Mass Transf..

[166] Rau M., Garimella S.V. (2014). Confined Jet Impingement with Boiling on a Variety of Enhanced Surfaces. ASME J. Heat Transf..

[167] Nishikawa K., Kusuda H., Yamasaki K., Tanaka K. (1969). Nucleate Boiling at Low Liquid Levels. Bull. JSME.

[168] Shukla M.Y., Kandlikar S.G. (2021). Influence of Liquid Height on Bubble Coalescence, Vapor Venting, Liquid Return, and Heat Transfer in Pool Boiling. Int. J. Heat Mass Transf..

[169] Ahn H.S., Sathyamurthi V., Banerjee D. (2009). Pool Boiling Experiments on a Nano-Structured Surface. IEEE Trans. Compon. Packag. Technol..

[170] Kong X., Zhang Y., Wei J. (2018). Experimental study of pool boiling heat transfer on novel bistructured surfaces based on micro-pin-finned surface. Exp. Therm. Fluid Sci..

